# Deletion of transketolase triggers a stringent metabolic response in promastigotes and loss of virulence in amastigotes of *Leishmania mexicana*

**DOI:** 10.1371/journal.ppat.1006953

**Published:** 2018-03-19

**Authors:** Julie Kovářová, Andrew W. Pountain, David Wildridge, Stefan Weidt, Frédéric Bringaud, Richard J. S. Burchmore, Fiona Achcar, Michael P. Barrett

**Affiliations:** 1 Wellcome Centre for Molecular Parasitology, Institute of Infection, Immunity and Inflammation, College of Medical, Veterinary and Life Sciences, University of Glasgow, Glasgow, United Kingdom; 2 Glasgow Polyomics, Wolfson Wohl Cancer Research Centre, Garscube Campus, College of Medical Veterinary and Life Sciences, University of Glasgow, Glasgow, United Kingdom; 3 Centre de Résonance Magnétique des Systèmes Biologiques, Université de Bordeaux, Bordeaux, France; Monash University, AUSTRALIA

## Abstract

Transketolase (TKT) is part of the non-oxidative branch of the pentose phosphate pathway (PPP). Here we describe the impact of removing this enzyme from the pathogenic protozoan *Leishmania mexicana*. Whereas the deletion had no obvious effect on cultured promastigote forms of the parasite, the Δtkt cells were not virulent in mice. Δtkt promastigotes were more susceptible to oxidative stress and various leishmanicidal drugs than wild-type, and metabolomics analysis revealed profound changes to metabolism in these cells. In addition to changes consistent with those directly related to the role of TKT in the PPP, central carbon metabolism was substantially decreased, the cells consumed significantly less glucose, flux through glycolysis diminished, and production of the main end products of metabolism was decreased. Only minor changes in RNA abundance from genes encoding enzymes in central carbon metabolism, however, were detected although fructose-1,6-bisphosphate aldolase activity was decreased two-fold in the knock-out cell line. We also showed that the dual localisation of TKT between cytosol and glycosomes is determined by the C-terminus of the enzyme and by engineering different variants of the enzyme we could alter its sub-cellular localisation. However, no effect on the overall flux of glucose was noted irrespective of whether the enzyme was found uniquely in either compartment, or in both.

## Author summary

*Leishmania* parasites endanger over 1 billion people worldwide, infecting 300,000 people and causing 20,000 deaths annually. In this study, we scrutinized metabolism in *Leishmania mexicana* after deletion of the gene encoding transketolase (TKT), an enzyme involved in sugar metabolism via the pentose phosphate pathway which plays key roles in creating ribose 5-phosphate for nucleotide synthesis and also defence against oxidative stress. The insect stage of the parasite, grown in culture medium, did not suffer from any obvious growth defect after the gene was deleted. However, its metabolism changed dramatically, with metabolomics indicating profound changes to flux through the pentose phosphate pathway: decreased glucose consumption, and generally enhanced efficiency in using metabolic substrates with reduced secretion of partially oxidised end products of metabolism. This ‘stringent’ metabolism is reminiscent of the mammalian stage parasites. The cells were also more sensitive to oxidative stress inducing agents and leishmanicidal drugs. Crucially, mice inoculated with the TKT knock-out parasites did not develop an infection pointing to the enzyme playing a key role in allowing the parasites to remain viable in the host, indicating that TKT may be considered a useful target for development of new drugs against leishmaniasis.

## Introduction

*Leishmania* are parasitic protozoa that cause a wide spectrum of diseases including a self-healing cutaneous form, a disfiguring mucocutaneous form and severe, often fatal, visceral leishmaniasis. The individual clinical manifestation and severity of the disease depends on the causative *Leishmania* species, immuno-susceptibility of the patient and various additional factors [1]. *Leishmania* are transmitted by sandflies, and they alternate in their life-cycle between two major stages, the promastigote—insect stage—and amastigote—mammalian stage. The different stages vary significantly in their morphology and biochemistry, each of them adapted to their specific environment. Better understanding of the parasites’ biology is required with focus on new drug targets and treatment development. As disruption of essential biochemical pathways has instant detrimental effects on parasites, understanding metabolism offers a means to enhance our capability to intervene against the leishmaniases.

The central carbon metabolism of *Leishmania mexicana* has recently been described using metabolomics approaches by Saunders *et al*. [2,3]. Promastigote culture forms use glucose as a major carbon source when available, fuelling glycolysis and subsequently the tricarboxylic acid (TCA) cycle [2]. Aspartate was also shown to fuel the TCA cycle although other amino acids were not directly assessed in that study [2]. Fatty acids have also been proposed as possible carbon sources for *L*. *mexicana* [4], but they are apparently utilised only in amastigotes, while promastigotes use exogenous fatty acids in lipid metabolism but not for energy generation [3]. Comparing metabolism in promastigotes and amastigotes led to the conclusion that metabolism in the latter stage is ‘stringent’ as glucose and other substrates were consumed at a comparatively lower rate and metabolic end products were generated in correspondingly reduced quantities [3,5]. The full extent of variation between the two major life stages remains unclear, however clear differences are evident [3,6–11]. One aspect of metabolism in trypanosomatid protozoa to have received particular attention is its unusual compartmentalisation with several pathways either totally, or partially, present within peroxisome-like organelles termed glycosomes, named because of the presence of many enzymes of the glycolytic pathway in this organelle. Other pathways, including the pentose phosphate pathway, fatty acid β-oxidation, nucleotide and ether lipid biosynthesis also localise to glycosomes [12], and understanding the role of these organelles in providing metabolic homeostasis is of considerable interest [13,14].

**Fig 1:**
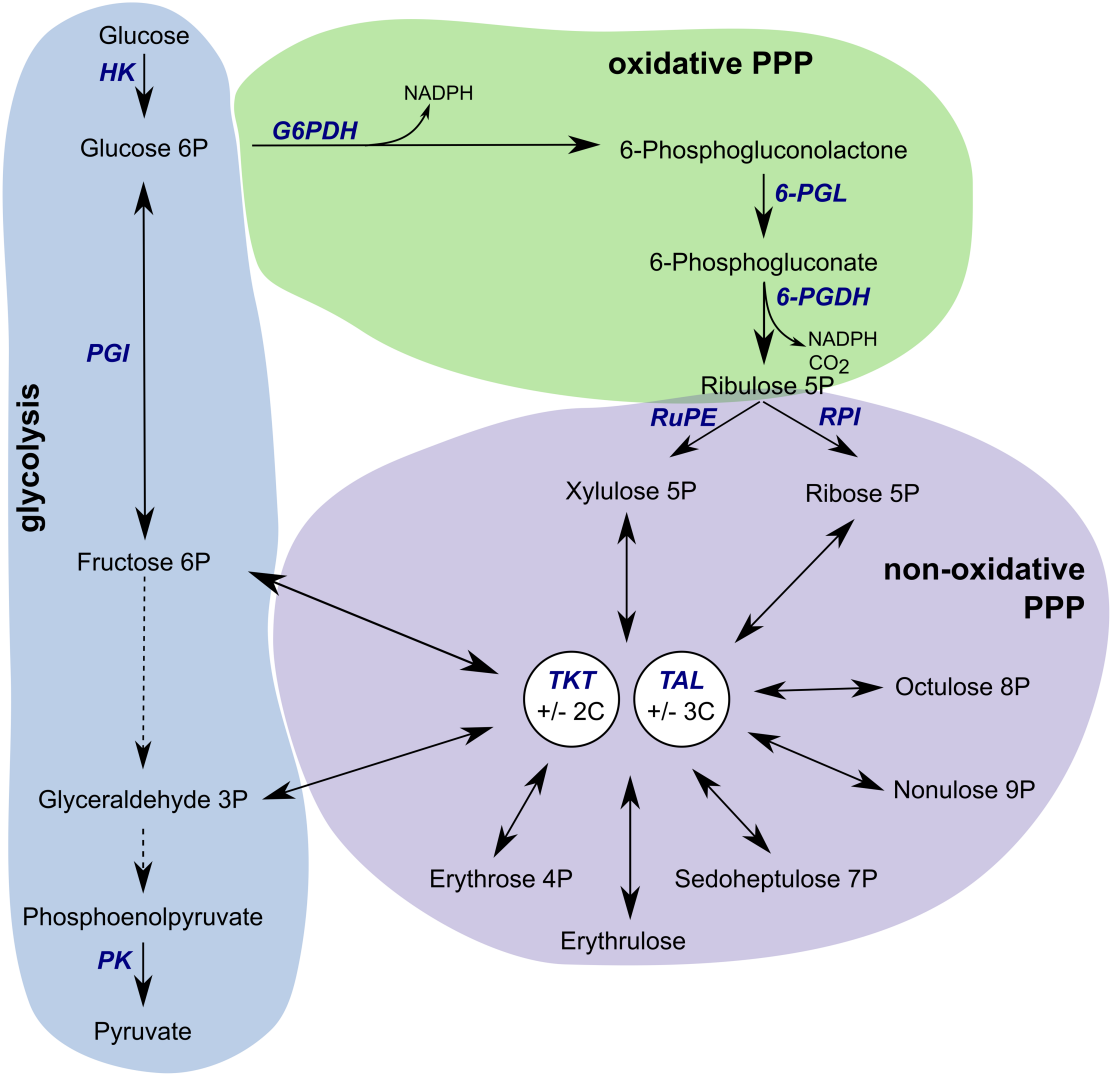
Pentose phosphate pathway. Glucose is phosphorylated into glucose 6-phosphate (G6P) by hexokinase (HK) and utilized further in glycolysis, shaded in blue, or channelled into the pentose phosphate pathway (PPP). In the oxidative branch of the PPP, shaded in green, G6P is converted via three subsequent steps comprising glucose-6-phosphate dehydrogenase (G6PDH), 6-phosphogluconolactonase (6-PGL), and 6-phosphogluocnate dehydrogenase (6-PGDH), resulting in the production of ribulose 5-phosphate, reduced NADPH and CO_2_. In the non-oxidative branch, shaded in lilac, ribulose 5-phosphate is converted by ribulose-5-phosphate epimerase (RuPE) to xylulose 5-phosphate or by ribose-5-phosphate isomerase (RPI) to ribose 5-phosphate. These and other sugar phosphates can be utilized by transketolase (TKT) and transaldolase (TAL) in reactions shuffling two or three carbons, respectively. Adapted from [35].

The pentose phosphate pathway (PPP) uses glucose 6-phosphate (G6P) to generate NADPH (Fig 1), the primary source of electrons in reductive biosynthesis and defence against oxidative stress, and also ribose 5-phosphate used in nucleotide metabolism [15]. The oxidative branch of the PPP comprises two dehydrogenases and 6-phosphogluconolactonase, and produces two molecules of NADPH and ribulose 5-phosphate (Ru5P). The following steps of the non-oxidative PPP consist of isomerisation reactions or transfer of carbons. Ru5P is converted by ribose-5-phosphate isomerase or ribulose-5-phosphate epimerase into ribose 5-phosphate (R5P) or xylulose 5-phosphate (X5P), respectively. These and other sugar phosphates, ranging from three to nine carbons in size, are used by transketolase (TKT) and transaldolase (TAL) which freely transfer two and three carbon units between sugar substrates. Some of their products (fructose 6-phosphate (F6P), glyceraldehyde 3-phosphate (GA3P)) can be fed back into glycolysis.

Both glucose-6-phosphate dehydrogenase (G6PDH) and 6-phosphogluconate dehydrogenase (6PGDH) were shown to be essential in bloodstream, but not procyclic *T*. *brucei* [13,16]. A G6PDH knock-out cell line in promastigote *L*. *major* suffered from severe growth defects and increased sensitivity to oxidative stress and antimonials [17]. The non-oxidative branch of the PPP has been subject to less study. Ribose-5-isomerase seems to be necessary for propagation of *L*. *infantum* and even parasites lacking just a single allele of the gene showed diminished infectivity in macrophages and in mice [18]. The discovery that TKT-like enzymes play an important role in a range of cancers [19–21] has reinvigorated interest in this enzyme. TKT transfers two carbon keto units between different sugar phosphates, ranging from three to seven carbons in size. TKT is not expressed in bloodstream form *T*. *brucei* although transaldolase (TAL), an enzyme classically considered to act in concert with TKT to shuffle carbons between sugars, is [22–24]. Knock-out of TKT in procyclic *T*. *brucei* had no effect on cellular phenotype, although substantial changes in various metabolites were detected by metabolomics analysis [22]. The crystal structure of TKT from *L*. *mexicana* has been resolved at 2.2 Å [25] and subcellular localisation studies revealed that around 70% of the activity was associated with the cytosol, with the rest compartmentalised within glycosomes [25]. The physiological role of TKT in *Leishmania* was not studied and it was speculated that the subcellular localisation depended on the context of a C-terminal peroxisomal targeting sequence [25].

Here, we present a study of a *L*. *mexicana* TKT knock-out (Δtkt) cell line. We examined the effects of TKT deletion on promastigotes and amastigotes in culture, and on their ability to infect macrophages and establish infections in mice. The promastigote cell line, as analysed by both targeted and untargeted metabolomics, revealed substantial changes in central metabolism. Notably, Δtkt cells exhibited changes reminiscent of the ‘stringent metabolic response’ described previously in amastigotes [3]. RNAseq analysis was performed to derive insight into regulatory mechanisms allowing parasites to adapt to metabolic perturbation. In addition, subcellular localisation of TKT and its regulation was scrutinized. Finally, we showed that parasites lacking TKT could not establish a virulent infection in mice.

## Results

### *L*. *mexicana* Δtkt cells are unaffected in growth as promastigotes but lose virulence in mice

**Fig 2:**
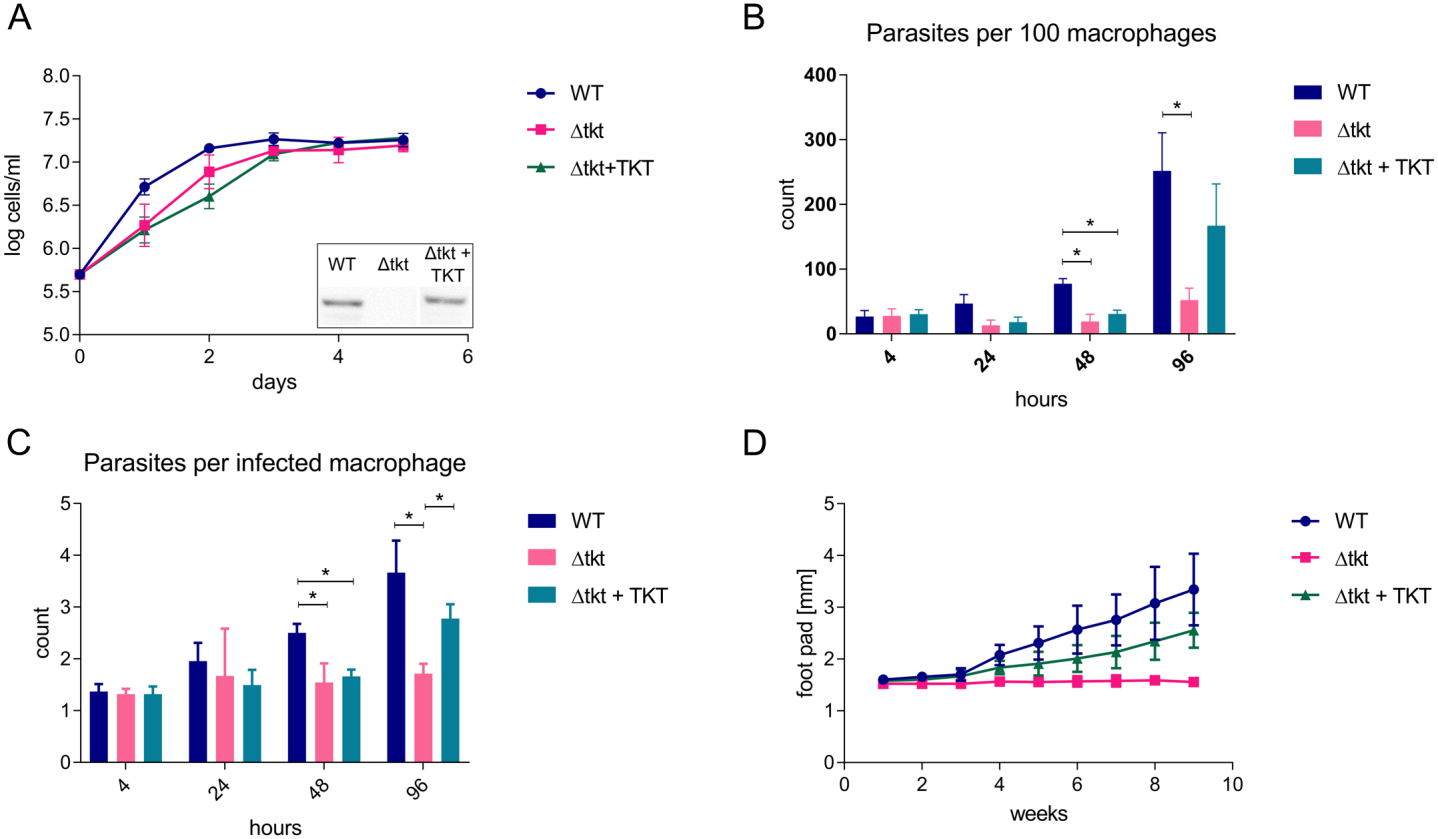
Growth of Δtkt cells *in vitro* and *in vivo*. (A) Growth of WT, Δtkt, and Δtkt + TKT promastigote cells in Homem medium over 5 days, n = 3. The inset shows Western blot analysis of WT, Δtkt, and Δtkt + TKT cell lines probed with α-TKT antibody. (B) Infection of THP-1 macrophages with *Leishmania*. After 4 hours of incubation the parasites were washed off and infection assessed at time points indicated. 10:1 parasite to host ratio, n = 4, * = p < 0.005. (C) Number of parasites per each infected macrophage was counted, n = 4, * = p < 0.005. (D) Development of foot pad lesions in BALB/c mice infected with WT, Δtkt, and Δtkt + TKT cells, n = 2, four and five mice were used per each group in the two respective replicates.

A TKT knock-out (Δtkt) cell line was constructed in *L*. *mexicana* by sequential replacement of both alleles of the gene with antibiotic resistance markers. PCR analysis revealed the gene was cleanly replaced by antibiotic resistance markers (S1 Fig). Promastigote cells did not suffer from any growth defect (Fig 2A). The Δtkt cell line was complemented with a copy of the TKT gene integrated into a ribosomal locus of the genome (Δtkt + TKT) and re-expression of TKT confirmed by Western blot (Fig 2A inset). This line was also unaffected for growth rate or morphology.

**Fig 3:**
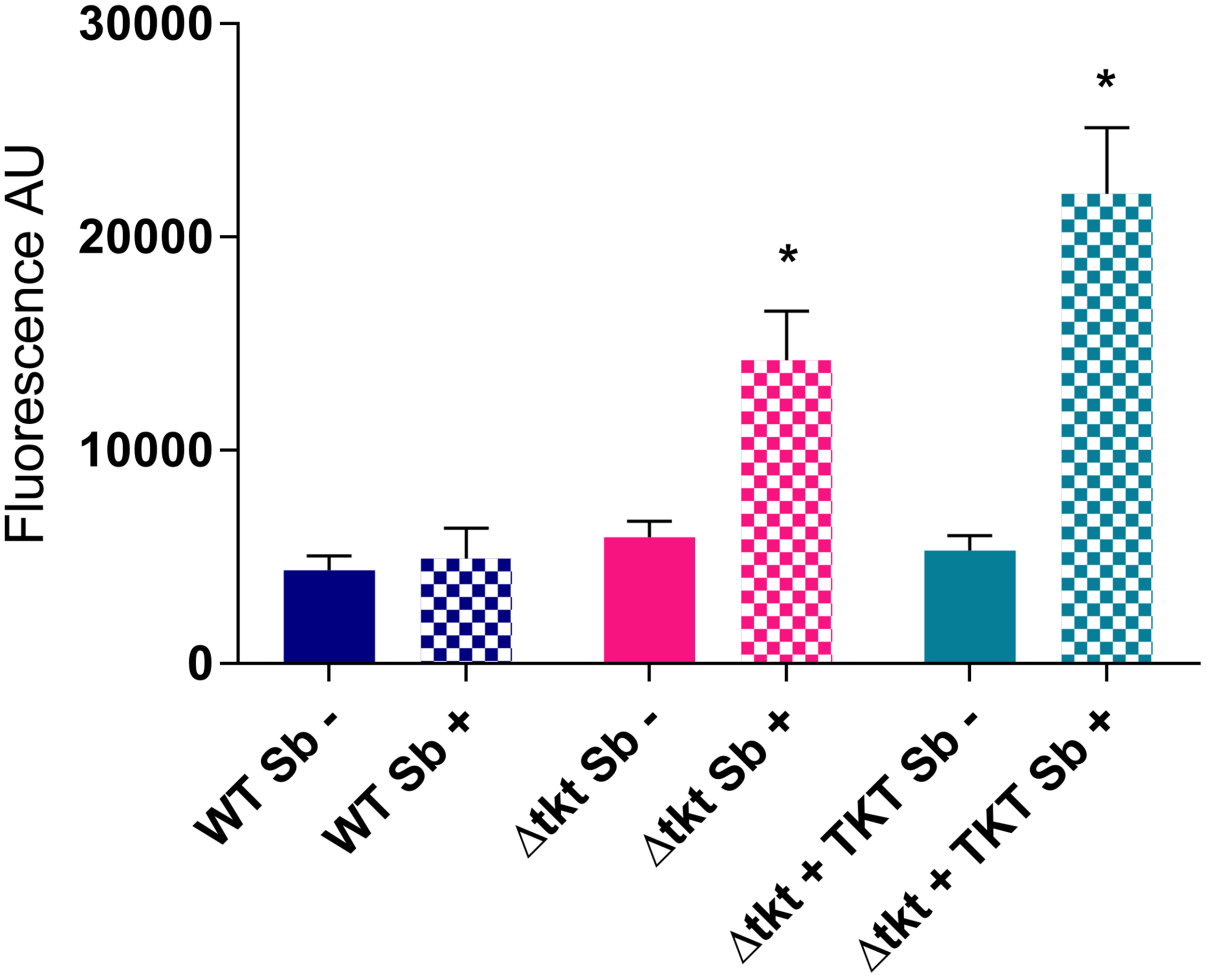
Measurement of reactive oxygen species by DCFDA. Reactive oxygen species inside cells were assessed directly by measuring DCFDA fluorescence. Whereas in untreated cells, ROS levels were slightly but significantly higher in Δtkt than WT, the difference was much more prominent after 8 h treatment with 200 μM potassium antimonyl tartrate, n = 6, * p < 0.0001.

The Δtkt cells were, however, significantly more sensitive to oxidative stress. Reactive oxygen species (ROS) can be directly detected with a fluorescent probe dichlorofluorescin diacetate (DCFDA) [26]. This probe showed that ROS were slightly but significantly higher in Δtkt than in WT (p = 0.004; Fig 3). After challenge with 200 μM Sb^3+^ for 8 hours, the difference was much more prominent with WT maintaining the same ROS levels, whereas Δtkt showed a three fold increase in ROS levels as measured by DCFDA (p = 1 x 10^−6^). Surprisingly, the phenotype was even stronger in Δtkt + TKT (five fold increase, p = 8 x 10^−8^).

When assayed with glucose oxidase added to culture medium as a constitutive source of H_2_O_2_, Δtkt were around twice as sensitive as WT (p = 0.01) while Δtkt + TKT re-expressor cells were partly rescued (1.5-fold decrease, p = 0.02). Another commonly used oxidative stress inductant, methylene blue, had much weaker differential effect on the cell lines (Table 1), although little is understood about its mode or site of action and additional effects. Sensitivity to other leishmanicidal compounds was also tested and Δtkt cells were more sensitive to all of those tested, albeit not significantly so, for paromomycin and antimonyl tartrate (Table 1). The addback Δtkt + TKT cell line did not recover to WT levels either in the DCFDA assay or with most of the drugs tested. Since most leishmanicidal drugs induce oxidative stress [26], it is probable that this enhanced sensitivity in Δtkt relates to the increased sensitivity to such stress.

**Table 1:** Sensitivity to oxidative stress inducing agents and other drugs.

IC_50_ values	WT	Δtkt	Δtkt + TKT
GOX [mU/ml] (n = 3)	2.32 ± 0.33	1.18 ± 0.27	1.53 ± 0.14
		p = 0.01	p = 0.019
Methylene blue [mM] (n = 4)	2.36 ± 0.34	1.88 ± 0.13	ND
		p = 0.04	
AmB [nM]	66.05	34.47	38.22
	63.03	24.2	34.15
Miltefosine [μM]	17.66	6.01	6.21
	22.24	5.59	6.02
Pentamidine [μM]	1.36	0.54	0.71
	1.4	0.69	0.63
Paromomycin [μM]	120.3	65.95	56.58
	128.5	90.87	51.66
Sb^3+^ [μM]	91.66	80.68	76.52
	106.9	73.23	96.46

In cases with more replicates, the number is indicated, and standard deviation and Student’s t-test was calculated. If only two replicates were performed, both values are indicated. GOX, glucose oxidase; ND, not determined.

We also transformed Δtkt promastigotes into axenic amastigotes and showed that transformation was possible but at significantly reduced efficiency when compared to WT amastigotes, and most importantly, the Δtkt cells did not multiply as an axenic culture (S1 Fig). The Δtkt line was also less able to establish infection in THP-1 macrophages (Fig 2B and 2C). Although initial infection was equal in all groups, significant increase was observed in WT (9.3 fold, p = 2.9 x 10^−4^) and Δtkt + TKT (5.5 fold, p = 0.0057) after 96 h, but no significant increase was observed in Δtkt (Fig 2B). Macrophages infected with Δtkt cells harboured in average 1.5 parasites per macrophage from the beginning up to 4 days, but these numbers increased over time for both WT and Δtkt + TKT (3.7 and 2.8, respectively; Fig 2C). To test the ability of Δtkt cells to establish an *in vivo* infection, BALB/c mice (n = 2, four and five mice per group) were infected with WT, Δtkt, and Δtkt + TKT parasites and the progression of cutaneous leishmaniasis lesions was assessed over time. Whereas mice infected with both WT and Δtkt + TKT cells developed typical footpad lesions after a few weeks, Δtkt cells did not cause any obvious lesions even after 9 weeks (Fig 2D). Δtkt parasites were recovered from the popliteal lymph nodes in small numbers of the infected animals after 9 weeks, but not after 20 weeks, although parasites were recovered from infections with WT and re-expressor cells in all cases. The data show that TKT is essential to *L*. *mexicana* in establishing mammalian infection.

### Untargeted metabolomics analysis of *L*. *mexicana* Δtkt promastigotes

**Fig 4:**
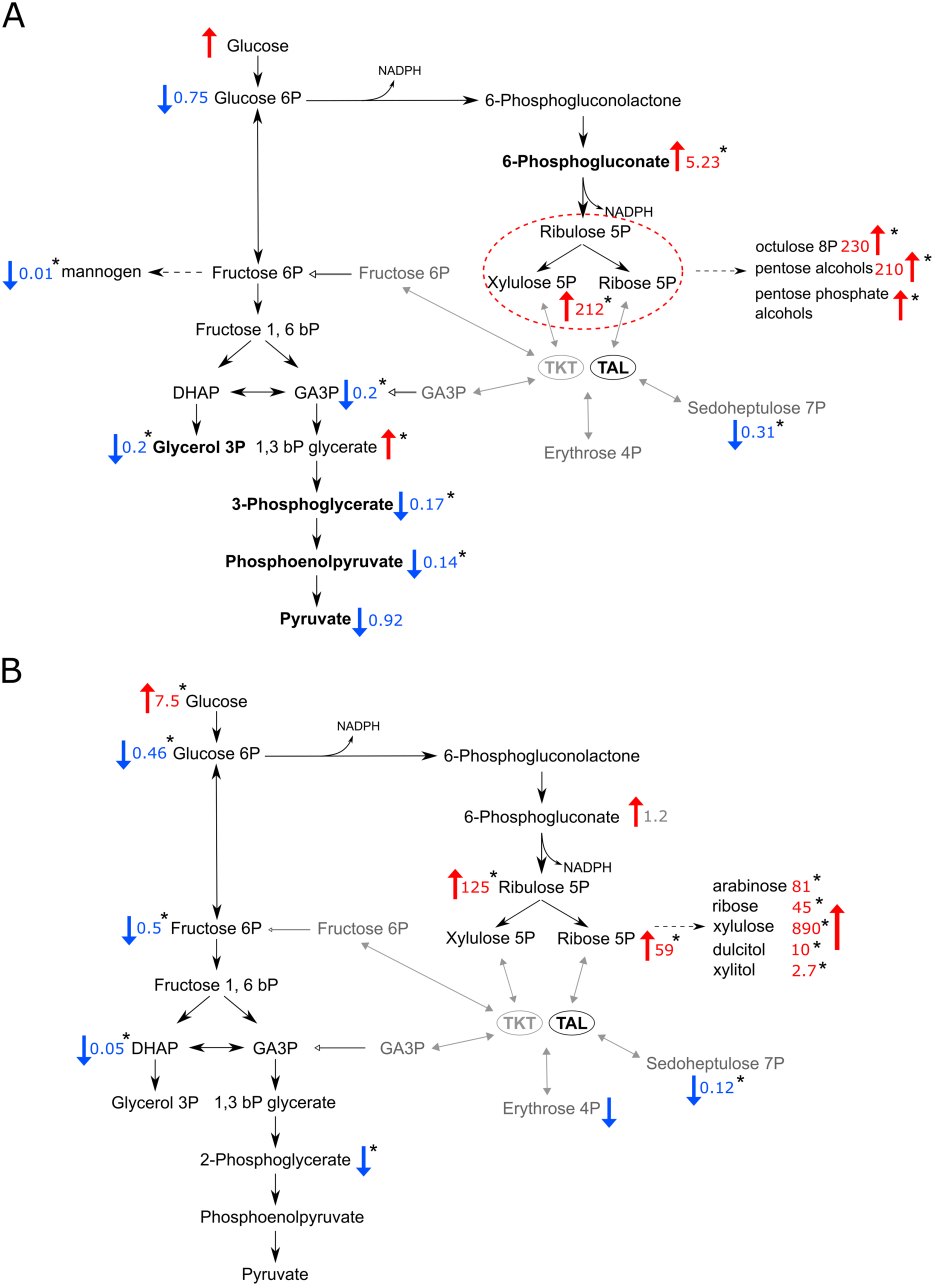
Relative changes in glycolysis and the PPP observed in metabolomic analyses. (A) Scheme of glycolysis and the PPP with indicated changes of metabolites detected by LC-MS analysis. Numbers are relative values in Δtkt compared to WT, red numbers and arrows indicate the fold increase, whereas blue indicates the fold decrease. Text and arrows in grey indicate steps missing after TKT depletion. Metabolites highlighted in bold were identified based on matches with respective standards, the rest are annotated based on mass and predicted retention time. (B) Similar scheme as in A, but showing GC-MS analysis. * p < 0.05.

Despite there being no discernible change to growth of Δtkt promastigote *L*. *mexicana*, LC-MS and GC-MS metabolomic analyses revealed profound changes to the cellular metabolome. Major changes to metabolites of the PPP were found. Metabolites occurring upstream of the TKT reactions, such as 6-phosphogluconate (6PG) and the pentose phosphates were significantly increased in abundance, whereas the product of TKT reaction, sedoheptulose 7-phosphate (S7P) was massively decreased (Fig 4, Table 2).

**Table 2:** Intensity of respective metabolites detected by metabolomic analyses. Values listed represent fold changes relative to WT.

	IC-MS/MS		LC-MS		GC-MS
cell line	Δtkt	Δtkt + TKT	Δtkt	Δtkt + TKT	Δtkt
G6P	0.80	0.28	0.75	0.77	0.46
F6P	0.70	0.25	-	-	0.5
6-PG	1.00	0.12	5.23 ^#^	0.58 ^#^	1.21
R5P	11.43	0.43	212	0.81	58.8
F1,6bP	0.60	0.40	-	-	-
2/3-phosphoglycerate	1.73	0.50	0.17 ^#^	0.65 ^#^	-
glycerol 3P	0.41	0.36	0.2 ^#^	0.59 ^#^	-
PEP	0.13	0.47	0.14 ^#^	0.67 ^#^	-
pyruvate	-	-	1.77 ^#^	1.75 ^#^	
alanine	-	-	0.89 ^#^	1.44 ^#^	0.15
Pen1P	1.80	0.91	-	-	-
M6P	0.25	0.20	-	-	-
fumarate	0.67	0.53	-	-	-
succinate	0.81	2.43	0.35 ^#^	1.64 ^#^	-
malate	0.69	0.67	0.68 ^#^	1.27 ^#^	-
ketoglutarate	1.18	0.68	0.51 ^#^	0.31 ^#^	-
citrate	1.57	1.15	1.38 ^#^	0.56 ^#^	-
cis-aconitate	1.00	1.00	1.41 ^#^	0.78 ^#^	-
orotic acid	1.00	1.00	1.06 ^#^	0.9 ^#^	-
E4P	23.45	0.72	-	-	-
S7P	0.07	0.18	0.31	0.77	0.12
G1P	0.60	0.18	-	-	-
C_18_H_32_O_16_	-	-	0.1	0.17	-
C_24_H_42_O_21_	-	-	0.1	0.18	-
C_30_H_52_O_26_	-	-	0.1	0.2	-
C_36_H_62_O_add_	-	-	0.15	0.25	-

IC-MS/MS, ion chromatography—tandem mass spectrometry; LC-MS, liquid chromatography—mass spectrometry; GC-MS, gas chromatography—mass spectrometry; G6P, glucose 6-phosphate; F6P, fructose 6-phosphate; 6-PG, 6-phosphogluconate; R5P, ribose 5-phosphate; F1,6bP, fructose 1,6-bisphosphate; PEP, phosphoenolpyruvate; Pen1P, pentose 1-phosphates; M6P, mannose 6-phosphate; E4P, erythrose 4-phosphate S7P, sedoheptulose 7-phosphate; G1P, glucose 1-phosphate.

^#^—metabolites identified based on matches with respective standards, all the other annotations in LC-MS are predicted.

The pool of accumulated metabolites also directs flux into additional reactions which were not detected in WT. For example, pentose phosphates are converted into their corresponding pentose alcohols or pentose phosphate alcohols. Additionally, octulose 8-phosphate (O8P) is accumulated, which is produced by a reaction of a pentose phosphate with a fructose 6-phosphate (F6P) or S7P by transaldolase, as can be verified by the labelling pattern (S2 Fig).

In addition to changes in the PPP metabolites and related levels of the glycolytic intermediates also decreased. Glucose accumulates while glucose 6-phosphate (G6P) is slightly decreased. The subsequent metabolites of glycolysis then diminish progressively, phosphoenolpyruvate (PEP) being decreased about seven fold compared to WT. Pyruvate is less diminished than PEP since it can be produced from malate or alanine via alternative metabolic pathways.

Interestingly, various predicted hexose oligomers (C_18_H_32_O_16_, C_24_H_42_O_21_, C_30_H_52_O_26_, C_36_H_62_O_31_) are massively decreased in Δtkt (Table 2). Most likely these all represent mannogen, the storage sugar polymer of *Leishmania* [27]. Other than these mannogen derivatives, levels of many metabolites that were perturbed in Δtkt cells returned to WT levels after TKT re-expression, or indicate a trend of rescue (Table 2).

### Central carbon metabolism is downregulated after TKT ablation

**Fig 5:**
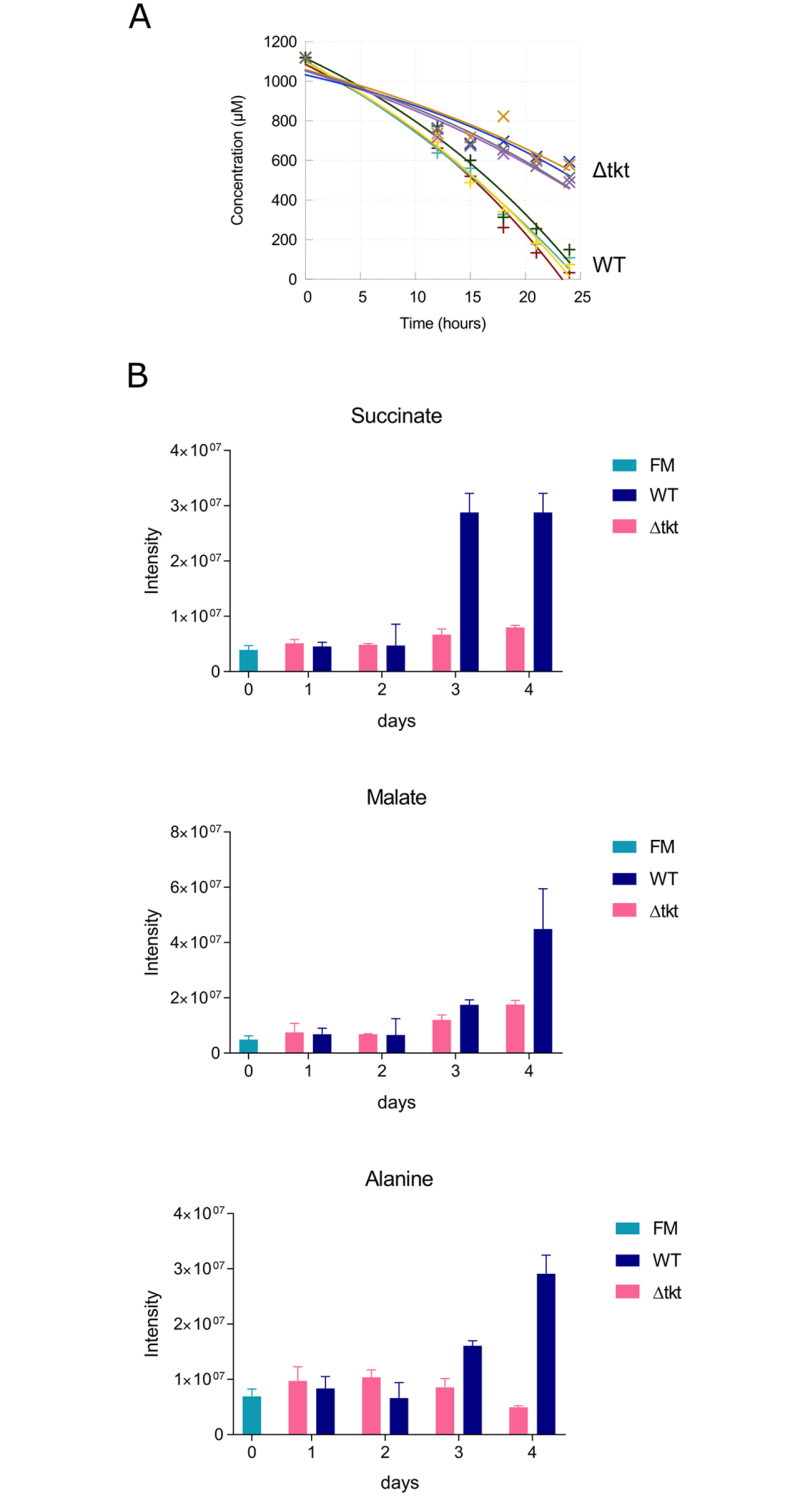
Central carbon flux is decreased in Δtkt. (A) Glucose consumption by WT and Δtkt cell lines. The graph indicates the concentration of glucose detected in spent media of the respective cultures, starting in Homem medium containing 1 mM glucose, n = 4. (B) Metabolic end products as detected in spent media of WT and Δtkt cultures by LC-MS, all three metabolites were identified based on matches with the respective standards. Growth rates were not significantly different. FM, fresh medium.

The decrease in abundance of glycolytic intermediates pointed to a downregulation of flux through glycolysis, in spite of TKT not playing a direct role in the pathway. Comparing glucose consumption in WT and Δtkt cells revealed the latter consumed significantly less glucose compared to WT, 3.9 +/-0.3 and 3.2 +/-0.3 nmol/min/10^8^ cells (p = 0.02), respectively (Fig 5A). Similarly, the production of major secreted high-energy metabolic end products was significantly decreased in Δtkt (Fig 5B). Succinate, malate and alanine were all produced and accumulated in the spent media of WT cells over four days, whereas these metabolites either did not accumulate at all, or only to a much lower extent in Δtkt. Succinate levels increased 12 fold in spent medium from WT, but only 2 fold in Δtkt, malate 7 fold in WT and 4 fold in Δtkt and alanine 3.5 fold in WT whilst it decreased to 70% of starting levels in Δtkt. Hence, the Δtkt cell line both consumes less glucose and produces less end products of metabolism than WT cells confirming a general decrease in glucose catabolism.

**Fig 6:**
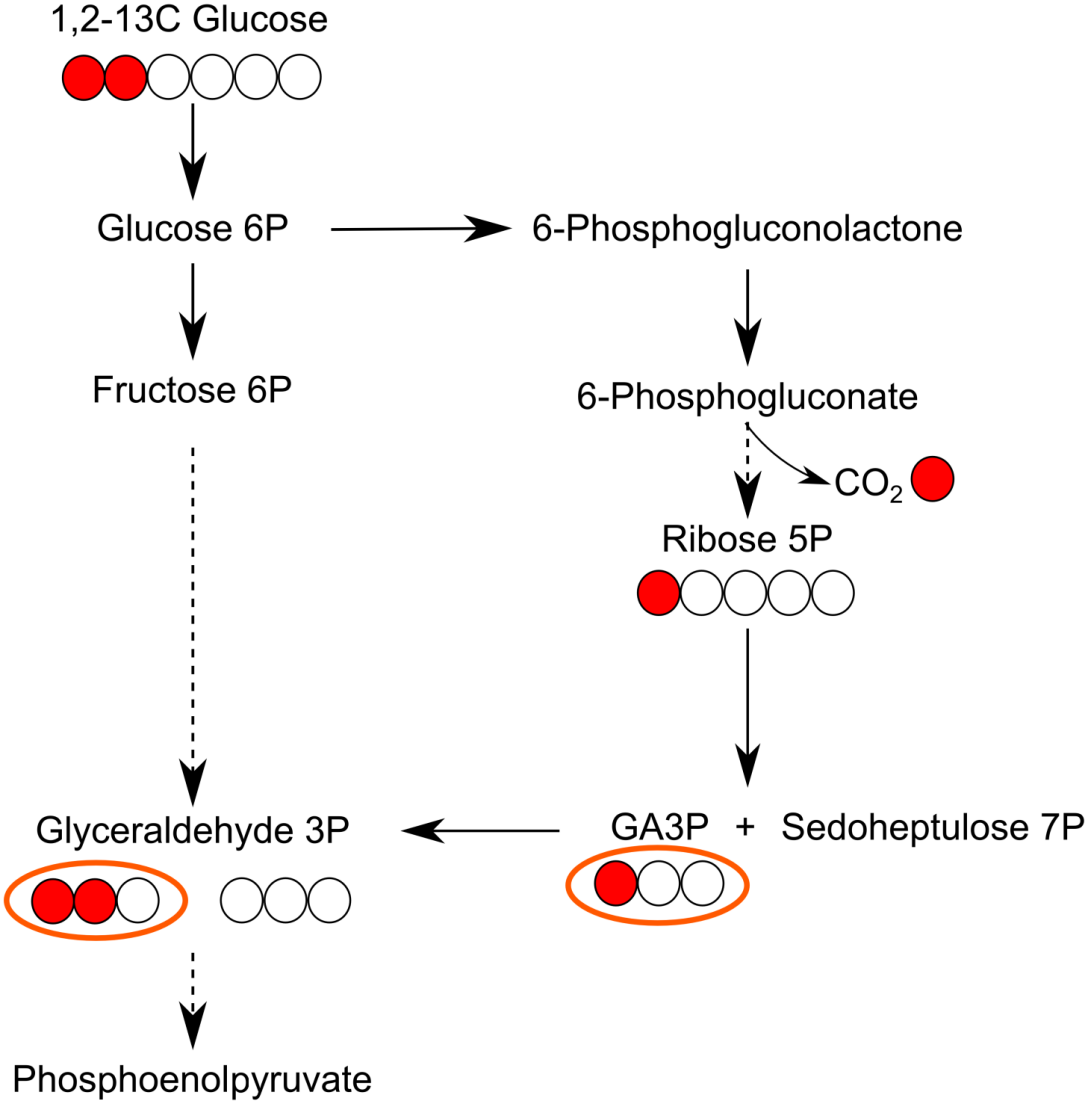
Scheme of the flow of carbon atoms from glucose through glycolysis and the PPP. If 1,2-^13^C_2_-glucose is used, products from glycolysis contain two or no carbons labelled, whereas in the PPP one carbon is cleaved as CO_2_, hence the three carbon products contain one or no carbons labelled. Based on Lee et al. [28]. Glyceraldehyde 3-phosphate (GA3P) used for the quantification is highlighted.

In spite of the fact that TKT is not part of the Embden-Meyerhof-Parnas glycolytic scheme, it does shunt carbon that has passed through the PPP back into glycolysis in concert with TAL. The Δtkt line should lose this ability. To assess what proportion of glucose passes via the PPP, we measured relative glucose flux through different routes using a method reported by Lee *et al*. [28]. The method involves feeding cells with 1,2-^13^C-glucose and then measuring the label in resultant three carbon containing derivatives (pyruvate and alanine). Glucose metabolised via the PPP loses the carbon from position-1 (cleaved by 6PGDH), hence the three carbon derivatives have only one carbon labelled, whereas if glucose is channelled into glycolysis, there will be either two or zero labelled carbons present in the three carbon products (Fig 6). The LC-MS analysis indicated that 14% of glucose enters the PPP in WT promastigote *L*. *mexicana*. Loss of this proportion of total carbon cannot account for the measured much higher loss of glycolytic flux, ruling out disruption of the PPP as a cause for decrease in glycolytic flux.

**Fig 7:**
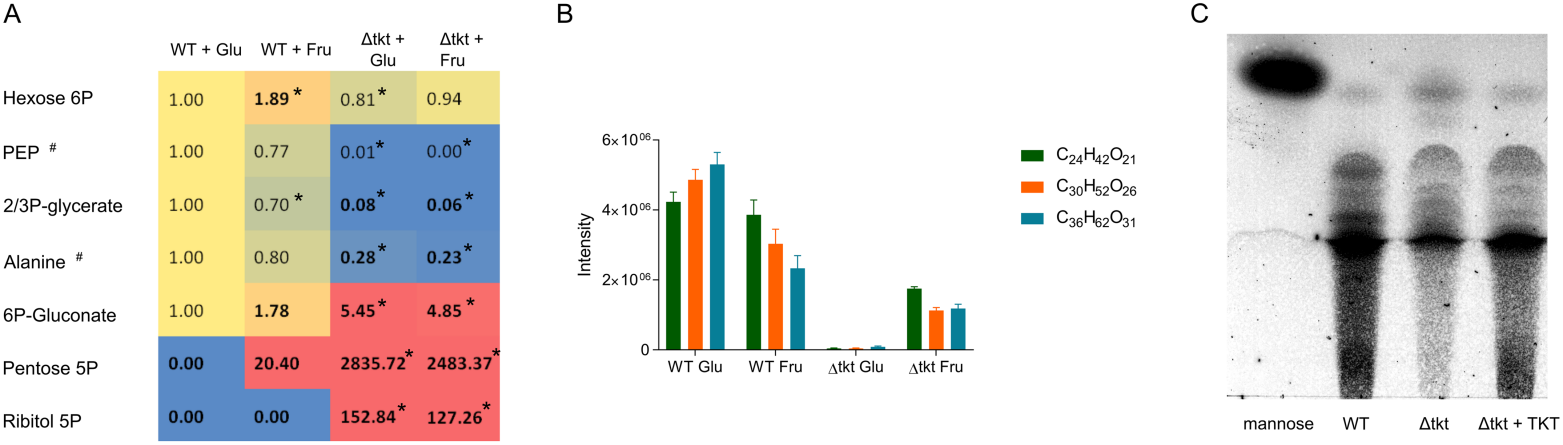
Mannogen is decreased in Δtkt. (A) Intermediates of glycolysis and the PPP as detected by LC-MS, when WT and Δtkt cells were grown in Homem medium containing either glucose (Glu), or fructose (Fru) as the main carbon source. Values in the first column (WT in glucose) are considered reference and the following values indicate relative change in intensity detected, n = 4. ^#^—metabolites identified based on matches with respective standards, all the other annotations are predicted. * p < 0.05. (B) Levels of sugar oligomers detected in WT and Δtkt cells grown in the presence of glucose or fructose, n = 4. Identity of these metabolites is predicted based on mass and retention time, however, the mass is very specific for sugar oligomers. (C) Mannogen as detected by HPTLC. Shown is one of three replicates.

An alternative explanation could be inhibition of PGI by 6PG, with a resultant block in glycolysis, as observed in yeast and Drosophila [29,30]. In this case, supplementation with fructose should rescue the phenotype (because fructose can be phosphorylated directly into F6P and channelled into glycolysis after the PGI reaction) [13]. To test this hypothesis, metabolomics analysis was performed with cells grown solely in the presence of glucose or fructose. However, fructose did not revert the phenotype and differences observed in the metabolome of Δtkt cells fed with glucose or fructose were minimal (Fig 7A). The only metabolite which was changed towards WT level was mannogen (represented by various hexose oligomers, Fig 7B).

**Fig 8:**
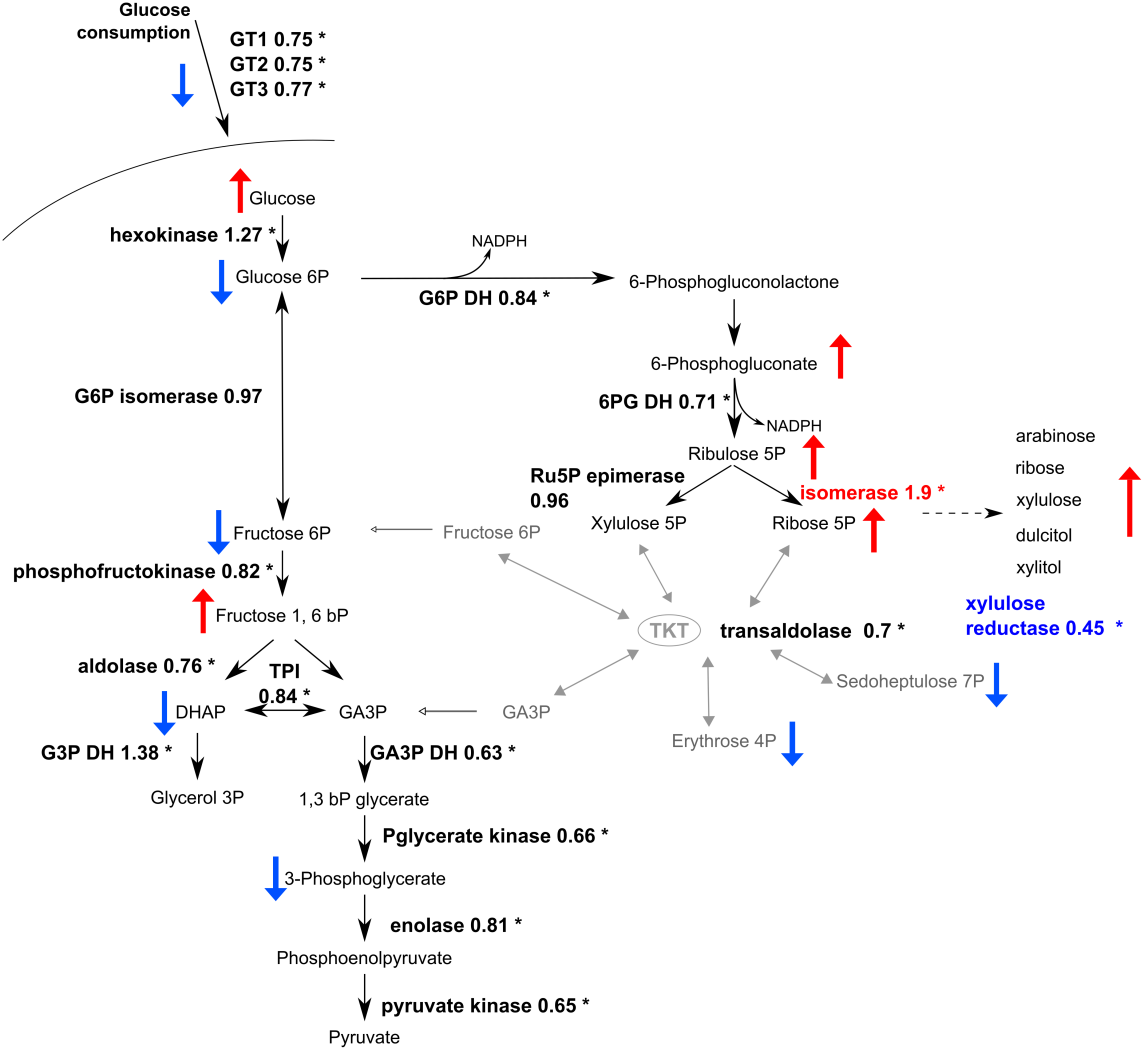
Relative shift in mRNA levels detected for the respective enzymes in glycolysis and the PPP by RNAseq analysis. Relative values detected in Δtkt when compared to WT are indicated. mRNA increased more than two fold is indicated in red, and decreases of more than two fold in blue, respectively. Red and blue arrows accompanying metabolites represent results of the GC-MS analysis, as depicted in Fig 4B. * p < 0.001.

Transcriptomic analysis revealed that RNA levels for genes encoding the glycolytic enzymes were changed less than two fold, although transcripts for the enzymes of the lower part of glycolysis were slightly but statistically significantly decreased (Fig 8, S3 Table). We also measured specific activities of several enzymes from cell extracts of WT and Δtkt cells. Fructose-1,6-bisphosphate aldolase activity was two fold lower in Δtkt (p = 0.0005; Table 3), whereas no significant differences were measured in activities of G6PDH, 6PGDH, hexokinase, or PGI between the two cell lines.

**Table 3:** Activities of selected enzymes as measured in WT and Δtkt cell lysates.

	WT	Δtkt	p-value
hexokinase	296 (±161)	429 (±139)	> 0.05
glucose -6-phosphate isomerase	338 (± 55)	232 (±28)	> 0.05
fructose -1,6-bisphosphate aldolase	202 (±15)	95 (±11)	0.0005
glucose-6-phosphate dehydrogenase	128 (±15)	107 (±25)	> 0.05
6-phosphogluconate dehydrogenase	131 (±20)	101 (±23)	> 0.05

Activity is calculated as nmol/min/mg protein, numbers in brackets indicate SEM, n = 4–5.

### Tracing glucose catabolism using U-^13^C-glucose

**Fig 9:**
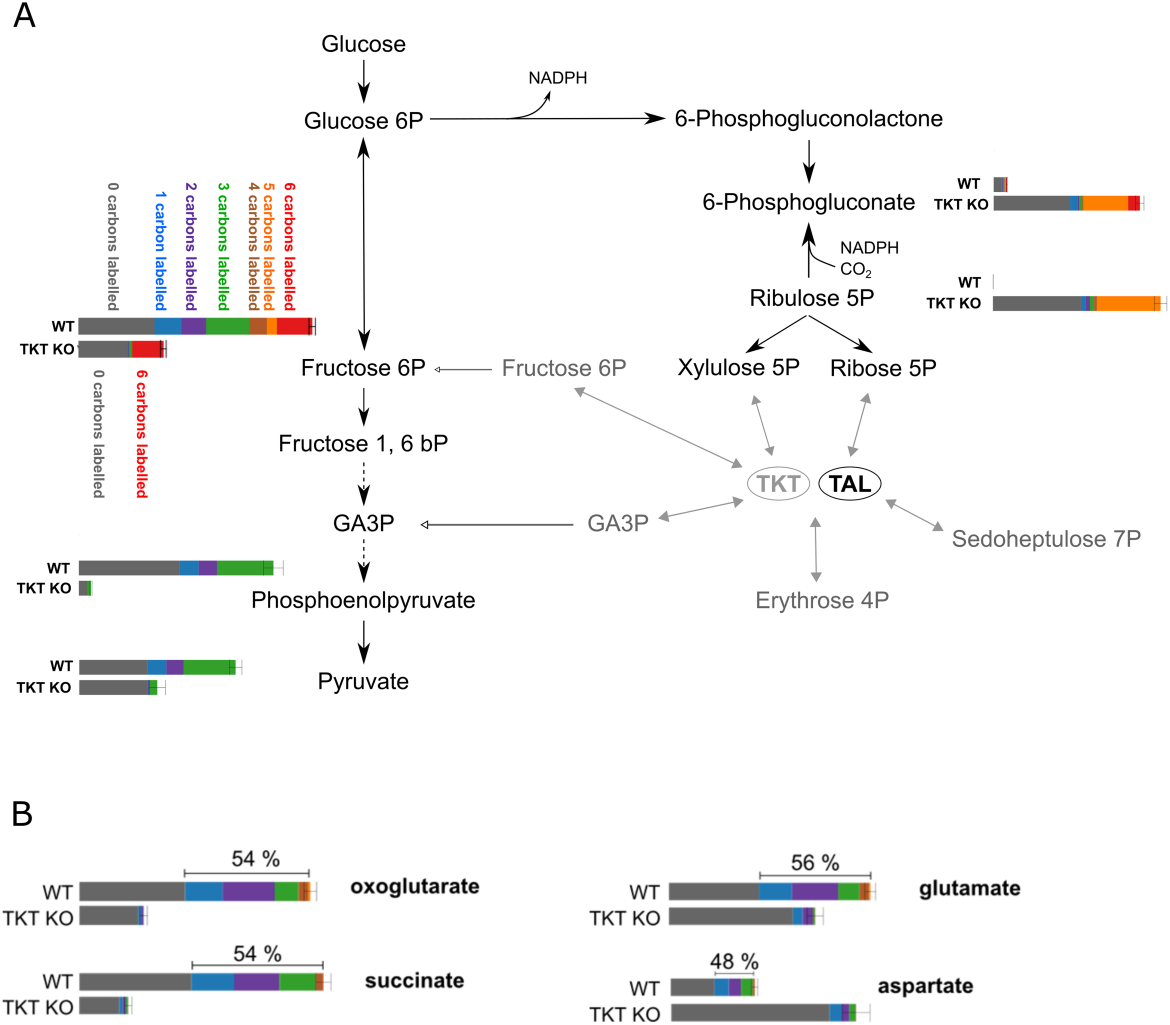
Difference in glucose utilisation in WT and Δtkt cell lines. Results of LC-MS metabolomics analysis when U-^13^C-glucose was used, 50% of glucose in the medium contained all carbons labelled, U-^13^C-glucose, and 50% was unlabelled glucose. Bars represent intensity of each respective metabolite detected. The part coloured grey is the non-labelled proportion, that labelled blue is one carbon, violet two carbons, green three carbons, orange four carbons and red six carbons labelled. (A) Metabolites of glycolysis and the PPP detected. (B) Intermediates of the TCA cycle and connected metabolites.

To learn more about the overall fate of glucose in WT and Δtkt cells, parasites were cultivated in medium containing 50% of glucose fully labelled (U-^13^C-glucose) and 50% unlabelled glucose. The labelling pattern observed in WT showed hexose phosphates containing anything between zero and six labelled carbons (33% 0C labelled, 11% 1C labelled, 10% 2C labelled, 19% 3C labelled, 7% 4C labelled, 5% 5C labelled, 15% 6C labelled) consistent with the carbon shuffling role played by the non-oxidative PPP (see Fig 1) resulting in production of F6P (Fig 9A). In contrast, practically all of the hexose phosphates in Δtkt cells were either labelled at all carbons or none (59% 0C labelled, 36% 6C labelled) confirming the key role played by TKT in carbon exchange.

In Δtkt cells 10% of 6PG contained one to four labelled carbons, 30% five labelled carbons, whereas 8% had six carbons labelled and 52% had no labelling, whereas WT comprised 25% one to four labelled carbons, 4% five labelled carbons, 8% six labelled carbons and 62% unlabelled (Fig 9A). The most plausible explanation for such an unusual pattern is that the high accumulation of Ru5P that accompanied the loss of TKT reverses the 6PGDH reaction, leading to production of 6PG from Ru5P while assimilating CO_2_ [31,32].

TCA cycle intermediates are of decreased overall abundance in Δtkt cells, and their labelling patterns differ markedly in the mutant when compared to WT. Whereas 50–60% of these metabolites are labelled with one through to all carbons in WT, over 90% of the total TCA cycle intermediates are unlabelled in Δtkt cells (Fig 9B). Of the measured 2-oxoglutarate, over 54% is labelled at one to five carbons in WT, whereas only 2% of the metabolite carried any label in Δtkt cells. Similar patterns were detected for succinate, glutamate and pyruvate. PEP consists of 29% three carbon, 9% two carbon, and 10% one carbon (2.5% is natural labelling) labelled in WT, whereas in the Δtkt line 28% is fully labelled and 72% unlabelled with no intermediate labelled-carbon states detected (Fig 9B). These results suggest that the TCA cycle is slightly decreased in Δtkt, but, more importantly, it is fed by carbon sources other than glucose.

**Fig 10:**
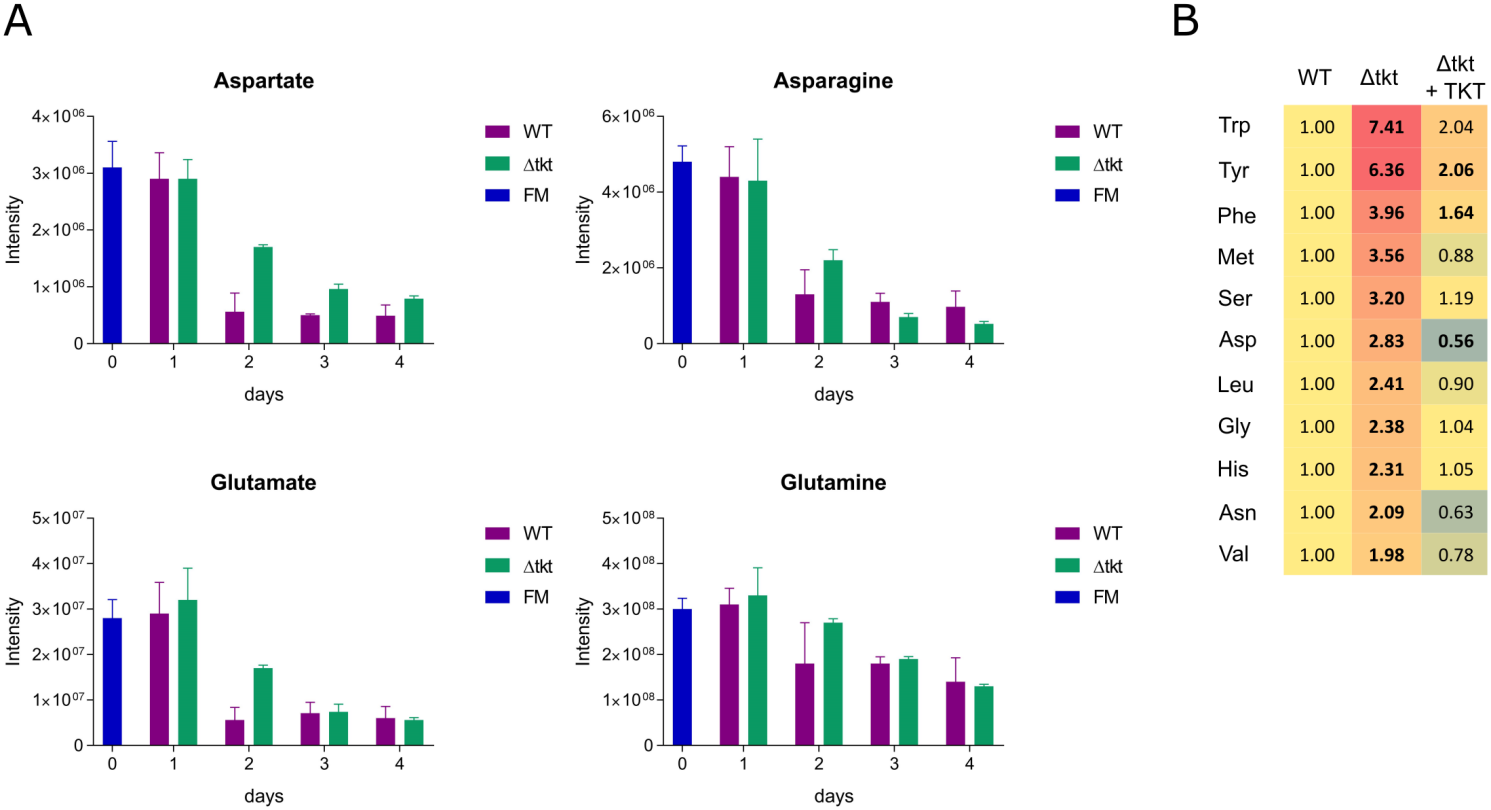
Relative levels of different amino acids measured in WT and Δtkt cells and their growth media. (A) Selected amino acids in fresh and spent media of WT and Δtkt cultures over four days of cultivation as detected by LC-MS analysis, FM—fresh medium, n = 4. (B) Intracellular levels of amino acids in WT, Δtkt, and Δtkt + TKT cultures detected by LC-MS. Intensity detected in WT is considered reference and the other values indicate relative abundance, n = 4. All the amino acids were identified based on matches with respective standards.

An untargeted metabolomics analysis of spent media was performed over 4 days of cultivation to determine whether other metabolites present in medium were used at enhanced rates in Δtkt as alternative sources of energy and carbon. Surprisingly, none of the amino acids was depleted more from Δtkt spent medium than in WT (except for small consumption of alanine in Δtkt, while it is produced in WT). Aspartate, glutamate and other amino acids were consumed, but either to the same extent in both cell lines or actually more in WT than in Δtkt (Fig 10A, S3 Fig). RNAseq analysis provided interesting complementary information on amino acid metabolism. RNA from six genes encoding members of the amino acid transporter family was around two fold increased in Δtkt, whereas mRNA of one family member was five fold decreased (S3 Table). Analysis of the intracellular metabolome of the two cell types indicates that eleven amino acids are of two fold or higher abundance in Δtkt cells (Fig 10B). However, neither transcriptome data (S3 Table), nor metabolome data (S1 Table), pointed to enhanced utilisation of those amino acids, hence, an accumulation of amino acids appears not to be accompanied by an increase in their utilisation.

There was no indication of enhanced utilisation of fatty acids nor any other nutrients by Δtkt over WT cells (S1 and S3 Tables). Δtkt cells, therefore are able to grow as quickly and to similar density as WT but consume fewer nutrients and create fewer partially oxidised end products of metabolism too. The situation is analogous to the so-called “stringent” response displayed by amastigote cells that also appear to reduce their consumption of metabolites when compared to promastigotes [3,5].

### Comparison of the transcriptome of Δtkt cells compared to WT

Metabolomics analysis indicated that beyond changes to the metabolite levels that could be directly attributed to loss of TKT within the PPP, there were more general changes to metabolism, including diminished flux of glucose via glycolysis and a switch to a more stringent metabolism resembling that in amastigote forms of the parasites. To assess whether changes in the steady state levels of RNA could contribute to this switch a transcriptomic analysis of WT, Δtkt and Δtkt + TKT was performed. Compared to WT, 150 genes were upregulated two fold or more and 156 downregulated to the same extent in Δtkt (p = 5 × 10^−5^, S3 Table). Since glucose catabolism was generally diminished we noted changes in transcripts associated with those pathways. Transcripts from the three principal tandemly arrayed glucose transporters LmGT1-3 [33] were all less abundant (75% levels compared to WT, p = 5 x 10^−5^). mRNA for a myo-inositol/proton symporter [34] was, by contrast, two fold upregulated. Changes of mRNA abundance for enzymes from glycolysis and the PPP are indicated in Fig 8, but for the majority of them the change was less than two fold. Of the PPP enzymes, mRNA of R5P isomerase gene was of almost two fold increased abundance in mutants (p = 5 x 10^−5^), while mRNA for xylulose reductase was of two fold lower abundance (p = 5 x 10^−5^). Generally, we conclude that changes in RNA levels play little or no role in diminishing glucose use in Δtkt mutants.

### Mannogen

The LC-MS analysis indicated depletion of hexose oligomers in Δtkt, which were partly restored after supplementation with fructose (Fig 7B). It is possible that these hexose oligomers are representatives of the poly-hexose mannogen which is a key metabolite in *Leishmania* [35,36]. We therefore, used HPTLC analysis which allows detection of all mannogen species within the cell. The result shown in Fig 7C indicated reduction of mannogen in Δtkt to around a half of the levels seen in WT (p = 0.029), and levels were partly restored in Δtkt + TKT (0.8 of WT, with no statistical difference p = 0.14).

**Fig 11:**
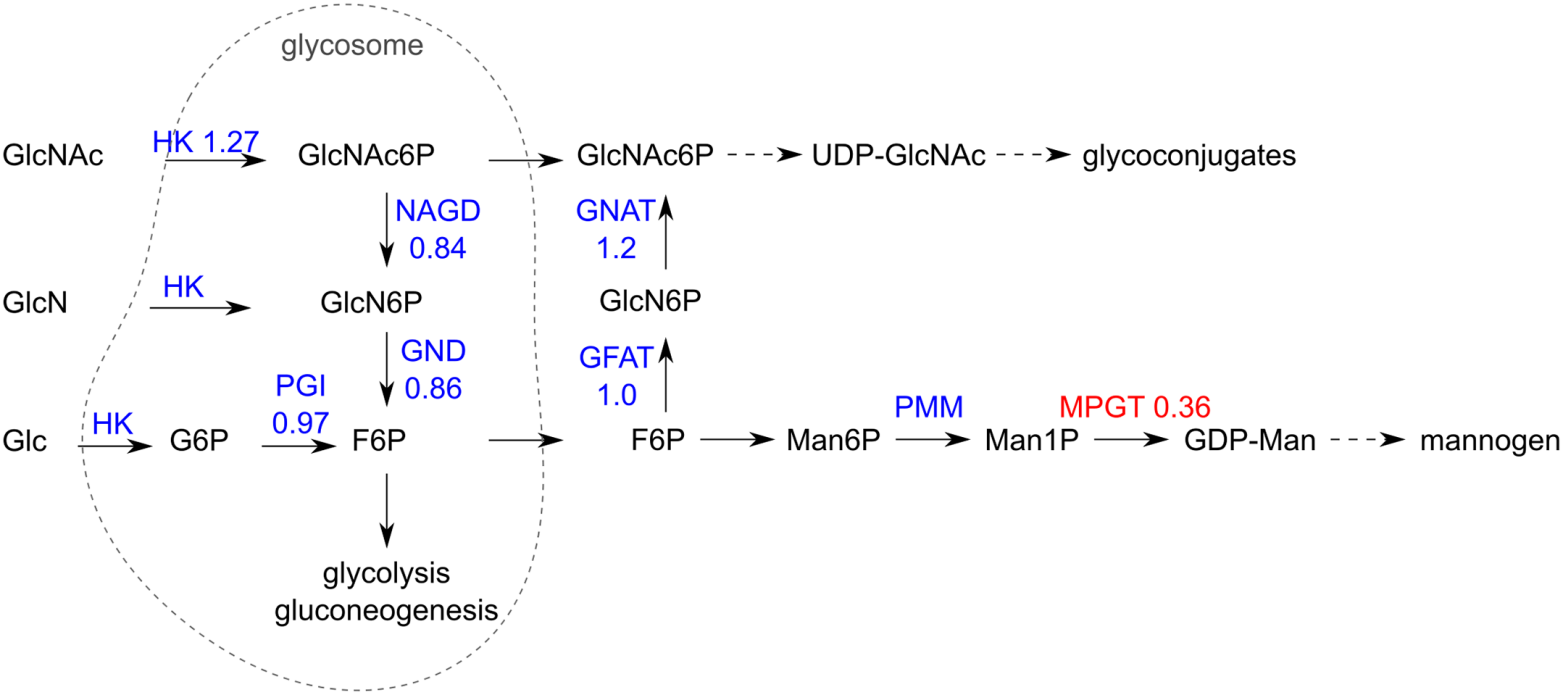
Scheme of mannogen biosynthesis. Blue and red colours indicate enzymes and their relative mRNA abundance in Δtkt when compared to WT. GlcNAc, N-acetylglucosamine; GlcN, glucosamine; Glc, glucose; GlcNAc6P, N-acetylglucosamine 6-phosphate; GlcN6P, glucosamine 6-phosphate; G6P, glucose 6-phosphate; F6P, fructose 6-phosphate; Man6P, mannose 6-phosphate; UDP-GlcNAc, uridine diphosphate N-acetylglucosamine; GDP-Man, guanosine diphosphate mannose; HK, hexokinase; PGI, glucose-6-phosphate isomerase; NAGD, N-acetylglucosamine-6-phosphate deacetylase; GND, glucosamine-6-phosphate deaminase; GNAT, glucosamine-6-phosphate acetylase; GFAT, glutamine:Fru6P aminotransferase; PMM, phosphomannomutase; MPGT, mannose-1-phosphate guanylyltransferase; dashed arrows indicate multiple enzymatic steps; based om Naderer, et al. [36] and Garami, et al. [70].

The transcriptome data indicated three fold decrease in the enzyme mannose-1-phosphate guanylyltransferase (EC 2.7.7.13, p = 5 x 10^−5^) of the mannogen biosynthetic pathway (Fig 11), but it fails to recover WT levels in the Δtkt + TKT cells.

### TKT localisation

**Fig 12:**
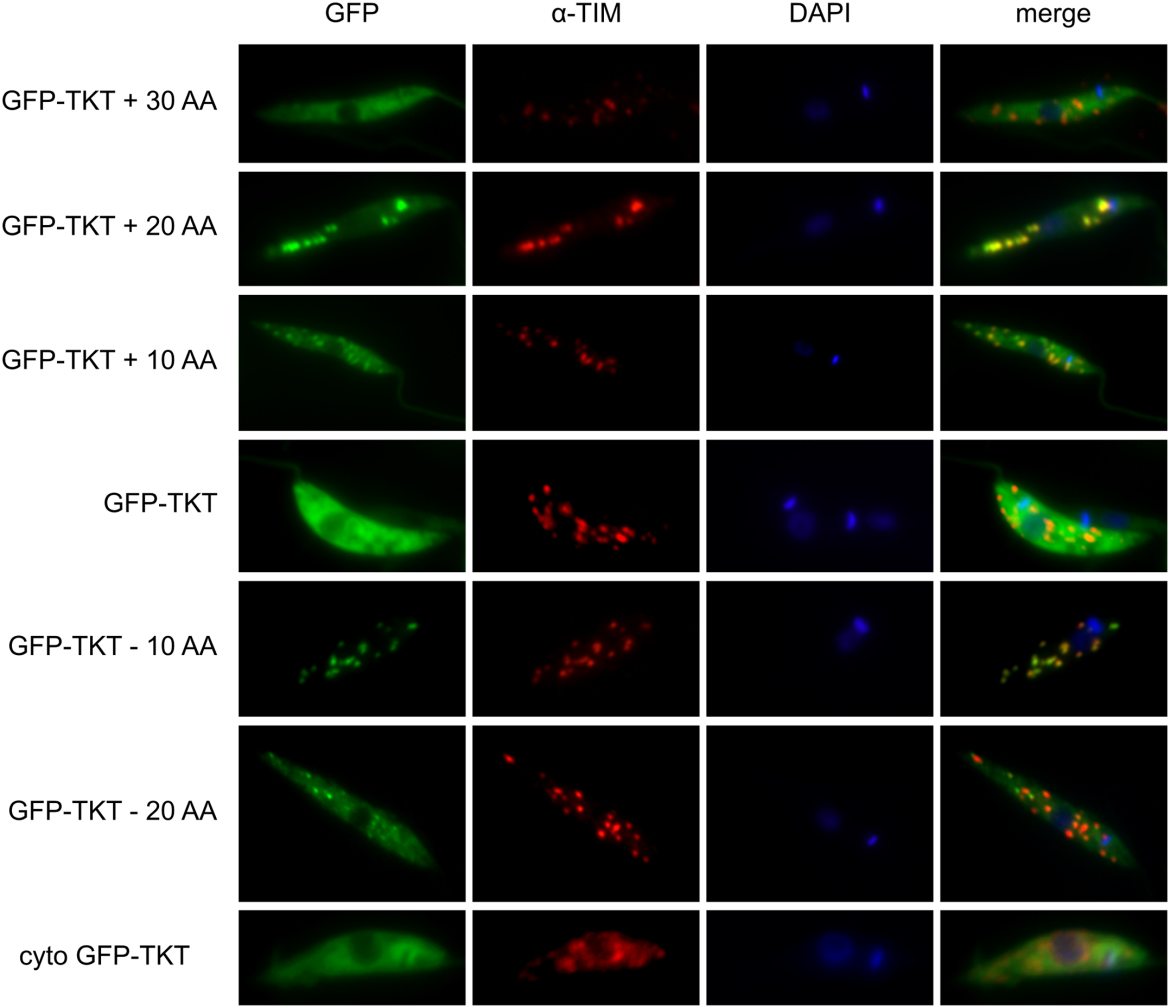
Immunofluorescence microscopy of cells transfected with all variants of altered GFP-TKT constructs. Shown from the longest at the top to the shortest at the bottom. Antibody against triosephosphate isomerase (TIM) was used as a glycosomal marker. The GFP signal corresponds to GFP-TKT complexes as verified by a Western blot analysis (S5C Fig).

TKT was previously shown to localise to both the cytosol and glycosomes in *L*. *mexicana*, with a majority of about 70% in the first compartment [25]. The C-terminus of TKT has a canonical PTS1 motif, SKM, but how this determines the dual localisation was unknown. The crystal structure of *L*. *mexicana* TKT had revealed the C-terminus to be unordered, prompting the suggestion that variability in this structure, perhaps induced by binding of regulators to the protein, could influence the positioning of the PTS and thus determine localisation [25]. We prepared a collection of episomal constructs with a GFP tag at the N-terminus of TKT and variants of the C-terminus of the protein either truncated at the C-terminus by 10, 20 or 30 amino acids, or elongated by 10 or 20 amino acids. The glycosomal targeting sequence was preserved in all of them and only the preceding sequence was altered. When analysed by immunofluorescence microscopy, these cell lines showed different subcellular localisation of TKT. Most strikingly, the TKT truncated by 10 AA was present solely in glycosomes, as confirmed by overlap with a known glycosomal marker triosephosphate isomerase (Fig 12, S4C Fig). The proteins elongated by 10 and 20 amino acids also showed higher signal overlapping with glycosomes, but there was still a significant part of the protein present in the cytosol. The other proteins (reduced by 20 AA or elongated by 30 AA) showed localisation similar to WT TKT or else a totally cytosolic TKT (from which the glycosomal targeting sequence ‘SKM’ was removed). The role of the C-terminal sequence in directing the protein is therefore proven, although how different proportions are targeted to different destinations remains unknown.

Obtaining a solely glycosomal TKT allowed us to further compare cells expressing TKT exclusively in this compartment. We transfected the GFP-glycoTKT (TKT minus 10 AA) plasmid into Δtkt cells, and in parallel a plasmid encoding a purely cytosolic TKT, i.e. one from which the PTS-1 sequence was removed. Surprisingly, these two cell lines did not show any significant differences. The growth rate of promastigote cells was the same and they were similarly sensitive to oxidative stress (S4 Fig). Furthermore, metabolomics analysis also revealed no major differences (S5 Fig, S1 Table) with metabolites of glycolysis and the PPP reaching the same levels, the only significant difference being observed in pentose 1,5-bisphosphate, which reached WT levels in Δtkt + cytoTKT, while dropped two fold in Δtkt + glycoTKT.

## Discussion

The PPP plays critical roles in NADPH production and generation of R5P for nucleotide metabolism in most organisms including parasitic trypanosomatids. A number of structural and pharmacological differences between enzymes of the pathway in trypanosomatids and their mammalian hosts has led to suggestions they may represent suitable drug targets [37,38]. Both G6PDH and 6PGDH have been shown to be essential in bloodstream *T*. *brucei* although dispensable in the procyclic stage [13,16]. G6PDH deletion also caused a strong defect in promastigote *L*. *major* growth, although effects in the amastigote stage have not been tested [17]. R5PI has been proposed as essential for *L*. *infantum* since its deletion from the genome was not possible [18]. TKT has recently emerged as a potential therapeutic target in cancer cells [19,21] where its role in protecting against oxidative stress has been proposed to be critical [20]. We show here that TKT is essential for *L*. *mexicana* virulence in mice, despite being dispensable in cultured promastigotes. TKT therefore offers a potential target for new drugs aimed at treating leishmaniasis, although there will be a time lag before suitable inhibitors are available for testing.

Although, Δtkt cells do not have any growth defect in the promastigote stage cultivated in rich Homem medium, the cells do develop enhanced sensitivity to oxidative stress and a variety of drugs used in therapy of leishmaniasis. These observations may be linked as most leishmanicidal drugs create oxidative stresses that contribute to their mode of action [26].

Despite there being no discernible growth phenotype in promastigote forms, metabolomics revealed that cellular metabolism was drastically altered in the knock-out cell line. A large accumulation of pentose phosphates occurred, as expected, since ribulose 5-phosphate, ribose 5-phosphate and xylulose 5-phosphate all fuel the non-oxidative branch of the pathway initiated by TKT. Correlated to this increase was a startling appearance of numerous alcohol derivatives of the pentoses. Moreover, Ru5P was converted back to 6PG by the reverse action of 6PGDH, since a substantial proportion of 6PG with five labelled carbons was detected in our heavy labelling experiment (with the unlabelled carbon fixed from CO_2_). This reaction will also perturb the NADP/NADPH balance in cells, which could explain the increased sensitivity to oxidative stresses. The direct product of TKT, S7P also decreased drastically in the mutant cell line as expected.

In addition to the alterations around the PPP itself, other metabolites involved in central carbon metabolism were also affected. An earlier study of carbon metabolism reported by Saunders and colleagues, revealed glucose to be the dominant carbon source with aspartate also entering the TCA cycle but other amino acids or fatty acids having little or no role [2]. Another study, however, indicated that utilisation of amino acids for mannogen biogenesis was highly dependent upon the presence or absence of glucose in the culture medium [39]. Following the distribution of carbons from labelled glucose here we confirmed that glucose is the main carbon source for the TCA cycle in WT parasites under standard culture conditions. Δtkt cells consume significantly less glucose than WT, but assessment of the composition of spent medium suggested that no individual metabolite replaces glucose as a predominant carbon and energy source in the Δtkt cell line. Aspartate, asparagine, glutamate and glutamine are all consumed substantially but only to the same or to a lesser extent in Δtkt cells compared to WT. A substantial amount of alanine is produced in WT, whereas it is partly consumed in Δtkt instead, providing a good example for the ‘stringent’ metabolism adaptations, where fewer resources are consumed and used more efficiently [3]. The Δtkt line did show increased levels of a number of amino acid gene transporter transcripts and also accumulated most amino acids, but no evidence for correspondingly higher levels of their catabolism was observed. However, the TCA cycle may be fuelled by a mixture of amino acids, in contrast to WT. Moreover, excreted end products of metabolism (malate, succinate and fumarate) are also less abundant in the Δtkt cells, and the mutant did not acidify the medium to the same extent as WT cells as evidenced by reduced colour change from red to yellow with the pH indicator in medium. The fact that the mutant line appears to consume less metabolic precursor substrates and also produces less secreted products is analogous to the ‘stringent metabolic response’ proposed for amastigote cells [3], possibly as a response to living in nutrient-limiting conditions, but also possibly to reduce perturbations to host metabolism which might stimulate anti-pathogen defences. An alternative to describing amastigotes and the TKT mutant as having ‘stringent’ metabolism would be to label metabolism of cultured promastigotes as ‘profligate’ since they appear to use unnecessarily high level of substrate and excrete excesses of partially oxidised end products of metabolism. Whether loss of TKT and the associated metabolic changes specifically trigger a stringent response, or such a response is a general response to ‘stress’ associated with loss of a key enzyme has yet to be resolved (it has been shown that reducing pH and increasing temperature also provoke a stringent response in promastigote *Leishmania* [5]).

Interestingly, flux via glycolysis is downregulated in Δtkt, but not as a direct consequence of the missing flux from the PPP, nor due to inhibition of PGI by accumulated 6PG as was previously shown in Drosophila and yeast [29,30]. In both *T*. *brucei* [13] and in promastigote *L*. *mexicana* we rule out this feedback loop since fructose, which enters glycolysis beneath the PGI step does not rescue flux through the glycolytic pathway. The drop in metabolites DHAP, GA3P and beyond is consistent with inhibition of fructose-1,6-bisphosphate aldolase, the enzyme that converts F1,6bP to DHAP and GA3P. Specific activity of aldolase was therefore measured in Δtkt cellular extracts and shown to be only half that of WT, while PPP enzymes G6PDH and 6PGDH were of comparable activity. In both plants and humans F1,6bP aldolase is inhibited by R5P and 6PG [40–42]. Both metabolites rise substantially in the Δtkt mutants, and the crucial amino acid residues are conserved in the protein sequence (S6 Fig), although whether there is similar inhibition of the leishmanial enzyme has not been tested. Inhibition of F1,6bP aldolase by accumulated PPP intermediates offers a plausible explanation for the observed decrease in glycolytic flux although direct evidence corroborating this is lacking.

We found that mannogen levels were two-fold decreased in the TKT mutant, and restored in the re-expressor cells. RNA sequence analysis revealed a decrease in mRNA levels of mannose-1-phosphate guanylyltransferase (MPGT) which may be associated with the reduction and may indicate an important role for this enzyme in the overall pathway to mannogen synthesis, as suggested previously [43]. Moreover, the hexose oligomers that may be related to mannogen are partly restored after supplementation of Δtkt with fructose in the medium. In contrast, WT cells grown on fructose contain lower levels of larger sugar oligomers, which suggests a substrate specific regulation of the pathway, although more information is lacking regarding regulatory mechanisms in mannogen synthesis or catabolism. Further work is required in order to explain the differences observed here.

By tracing the isotope distribution in three-carbon products derived from glucose labelled either in position-1 or in positions-1 and -2 we calculated that 14% of glucose flux is via the PPP in promastigote *L*. *mexicana*, similar to the 11% reported previously [44] based on measurements of incorporation of radiolabelled glucose into DNA. In other organisms, glucose flux via the PPP has been reported between 10% to 41% in *T*. *cruzi* [45,46], 7% in healthy humans [47], 5.7% in human hepatoma cells [28], net flux being dependent upon cellular requirements. Comparing the labelling patterns of products of glucose metabolism in WT and Δtkt cells revealed profound differences. Without TKT, the extensive and rapid shuffling of carbons seen in WT disappears, confirming the key role that TKT plays in shuffling carbons between different sugar phosphates in the non-oxidative PPP [48].

The discovery that TKT is essential for long-term viability of amastigotes in mice indicates that the enzyme may be viewed as a possible drug target. As efforts to develop inhibitors of mammalian TKT are underway, a vein of small molecules to test for activity against the *Leishmania* enzyme may become available. Mannogen depletion may be responsible for loss of virulence since in Δtkt, mannogen levels are decreased and this polymer has previously been reported to be essential for amastigote survival [35]. Accordingly, in the Δtkt + TKT infectivity is restored, and mannogen levels recover. Contribution of other factors cannot be excluded, including defence against oxidative stress.

Since the PPP is a major source of cellular NADPH, it is also possible that perturbing the pathway disrupts the cellular redox balance to a lethal extent. In addition to diminished overall production of NADPH in the PPP, the accumulation of ribulose 5-phosphate stimulates the 6PGDH reaction to run in reverse and consumption of this key redox metabolite will exacerbate the problem. Promastigote Δtkt cells produce around three fold more ROS than WT, but amastigotes are exposed to more stress than promastigotes and this defect may be more prominent. Moreover, NADPH is also necessary for reductive biosyntheses, for example of fatty acids and ergosterol [49,50]. Direct toxic roles for products accumulating due to loss of the TKT reaction, or ensuing steps in amastigotes cannot be excluded. For example, octulose 8-phosphate is a metabolite not previously described in *Leishmania* and its production is greatly increased in TKT defective cells, as is a range of other pentose sugars and their alcohol derivatives. Loss of other metabolites e.g. S7P might also play a role.

We also addressed the question of the dual localisation of TKT where the same enzyme has a primary localisation within the cytosol but a minor part is also found in glycosomes, presumably due to the presence of a canonical C-terminal type-1 peroxisomal targeting tripeptide motif, SKM. Removal of the SKM motif led to all of the enzyme being found in the cytosol. To test whether the structurally disordered sequence preceding the PTS-1 motif might be involved in altering presentation of that sequence, we created cell lines expressing the protein with altered C-termini. When we added stretches of either 10 or 20 amino acids to the sequence prior to the SKM motif, the expressed protein was now found primarily in the glycosome. However, removing 10 amino acids, also led to a protein found primarily in the glycosomes, whilst removal of 20 or addition of 30 amino acids created a protein that was mainly cytosolic. The data are not compatible with the simple notion of a retractable C-terminus (where it would be expected that longer versions would be constitutively glycosomal and shorter versions constitutively cytosolic based on the ability of the protein to bind to PEX5 in the cytosol) hence the molecular mechanism of dual localisation remains uncertain.

Irrespective of the mechanism determining dual localisation, metabolomics analysis revealed that the cellular metabolome of *L*. *mexicana* promastigotes was similar in cells expressing solely cytosolic or solely glycosomal versions of the enzyme and sensitivity to oxidative stress was also the same. These results are consistent with the semi-permeable nature of glycosomes whereby the distribution of most small metabolites is not affected by the presence of the glycosomal membrane [51]. However, these analyses were all performed in promastigote cells cultivated in rich medium and important roles associated with the enzyme’s subcellular localisation in amastigotes, or in other stages of the parasite in its natural life cycle are not known.

In summary, we show that TKT is involved primarily in the shuffling of carbon atoms between different carbohydrates in *Leishmania*, and that this activity may contribute to mannogen biosynthesis. It appears to have a role in protecting promastigotes against oxidative stress. Loss of the enzyme stimulates a shift towards a reduced metabolic rate redolent of the ‘stringent’ response shown by amastigote forms of the parasite. The C-terminus of the enzyme determines its subcellular localisation, although a mechanism for how it is dually localised to glycosomes and cytosol is not known. In promastigotes the enzyme’s distribution does not influence overall metabolism of the cells. Most intriguingly, TKT knockout cells were not able to establish infection in mice, although cells re-expressing the enzyme were, indicating the TKT is essential to *Leishmania* and a possible target for chemotherapy.

## Materials and methods

### Ethics statement

All animal experiments were performed in accordance with the Animals (Scientific Procedures) Act 1986 and the University of Glasgow care and maintenance guidelines. All animal protocols and procedures were approved by The Home Office of the UK government and the University of Glasgow Ethics Committee under Project license PPL 60/4442.

### *Leishmania* cultivation

*Leishmania mexicana* M379 cells were cultivated in Homem medium (GE Healthcare) supplemented with 10% foetal calf serum (FCS, Thermo Fisher Scientific) at 26°C. Δtkt cell culture was supplemented with 25μg/ml nourseothricin (Sigma-Aldrich) and 25μg/ml hygromycin (Roche), integrated re-expressor cell line Δtkt + TKT with additional 25μg/ml puromycin (Sigma-Aldrich). Cell lines with episomal vectors were cultivated in additional 25μg/ml G418 (Sigma-Aldrich). Amastigote cells were cultivated in Schneider’s Insect Medium (Sigma-Aldrich) supplemented with 20% FCS (Thermo Fisher Scientific) and 15 mg/l hemin (Sigma-Aldrich), at 32°C, pH 4.5, and 5% CO_2_ [52].

### Generation of the Δtkt line

LmTKT alleles were replaced sequentially with hygromycin B phosphotransferase (HYG) and streptothricin acetyltransferase (SAT) genes, encoding resistance markers for the antibiotics hygromycin and nourseothricin, respectively. The gene deletion construct for nourseothricin selection was based on the *Leishmania* knockout construct JPCM5 CPA-sat (pGL520; kindly provided by Prof. Jeremy Mottram, University of York), a derivative of pXG-63-hyg (PMID 8650210). Flanking sequences upstream (0.4 kb) and downstream (0.6 kb) of the transketolase ORF were amplified by PCR using oligonucleotides that incorporated restriction sites suitable for subsequent insertion into the polylinker regions flanking the SAT gene and DHFR-TS untranslated regions of pGL520. The 0.4 kb upstream fragment was amplified with oligonucleotides 5’-TAGCGTCGACTGTGCTTGTGGGTGAGGGCG and 5’-TAGCAAGCTTGGCCGCTTCGCACCACACGA, restriction sites for *Sal*I and *Hind*III are underlined. The 0.6 kb downstream fragment was amplified with oligonucleotides 5’-TAGCCCCGGGTGCTCCGAAACGTGAGGAAT and 5’-TAGCAGATCTACTTCCTTGCCCTTCCGATA, restriction sites for *Sma*I and *Bgl*II are underlined. The hygromycin B-phosphotransferase resistant plasmid was generated by replacing the streptothricin acetyltransferase resistance gene, with the hygromycin B resistance gene from pGL345 (PMID 8650210). The knockout cassettes used for transfection was released by *Hind*III and *Bgl*II and gel purified using the QIAquick Gel Extraction Kit (Qiagen). Clonal-like population of parasites resistant to hygromycin and nourseothricin were analysed by PCR and Southern Blot.

### Ribosomal expression

For stable integration into the ribosomal locus, a derivative of pSSU-int [53] was used. The transketolase ORF was amplified using oligonucleotides 5’-TAGACTCGAGCTTCGCCTCTCTTCGTCGCCCT and 5’-TAGAGCGGCCGCCGCCTCTTCCGGTGTCATTC, restriction sites for *Xho*I and *Not*I are underlined. Clonal-like population of parasites resistant to puromycin were analysed by PCR.

### Animal infections

For mouse infection assay, BALB/c mice were inoculated with 2 x 10^6^ promastigote cells from cultures in stationary phase, in the right rear foot pad and the size of ensuing lesions was measured weekly. At the end of the experiment popliteal lymph nodes were removed, placed into Homem medium at 26°C, and recovery of the parasites was assessed. Presence/absence of the TKT gene in these recovered parasites was confirmed by PCR.

### Alamar Blue Assays

Alamar Blue Assays were performed as described previously [54,55]. Briefly, the assay was started with promastigotes at 5 x 10^5^ cells/ml cultivated in the presence of respective compounds at desired concentrations (glucose oxidase, amphotericin B, methylene blue, miltefosine, paromomycin, potassium antimonyl tartrate (all Sigma-Aldrich), pentamidine (May & Baker)) after 72 h cultivation resazurin (Sigma-Aldrich) was added to final concentration of 49 μM and after additional 48 h incubation, absorbance was measured with fluorescence spectrometer FLUOstar OPTIMA (BMG Labtech).

### Measurement of intracellular ROS in *Leishmania* promastigotes

Intracellular ROS was measured using dichlorofluorescein diacetate (DCFDA) as previously described but with modifications [26]. Mid-log phase promastigotes were incubated for eight hours with or without 200 μM potassium antimonyl tartrate (Sigma-Aldrich). Parasites were then precipitated by centrifugation, washed once with PBS then resuspended in HEPES-NaCl assay buffer (21 mM HEPES, 137 mM NaCl, 5 mM KCl, 0.7 mM Na_2_HPO_4_, 6 mM glucose at pH 7.4) to a density of 7.5 x 10^7^ cells/ml with 4 μM DCFDA (Sigma-Aldrich). Two 200 μl aliquots taken from each sample were incubated for 40 min at 25 °C before measuring fluorescence on a FLUOstar Optima microplate reader (BMG Labtech) with excitation wavelength of 485 nm and emission wavelength of 520 nm. The average of these technical replicates was calculated, from which was subtracted a background value, determined as the average measurements of five wells containing only HEPES-NaCl buffer with 4 μM DCFDA.

### Infection of THP-1 cells

Human monocyte-lineage THP-1 cells were routinely cultured in RPMI (Gibco) supplemented with 10% FBS (Gibco), 2 mM glutamine and 100 units/ml penicillin/0.1 mg/ml streptomycin (Sigma-Aldrich) (complete RPMI) at 37 °C, 5% CO2. Prior to infection, THP-1 cells were subject to differentiation for 24 hours with 40 ng/μl phorbol 12-myristate 13-acetate (PMA, Sigma-Aldrich), after which cells were adherent and PMA was removed by washing with serum-free RPMI. Stationary-phase *L*. *mexicana* promastigote cultures were precipitated by centrifugation and washed twice in serum-free RPMI, prior to resuspension in complete RPMI. Infections were carried out in a 10:1 parasite:host cell ratio for four hours at 32 °C, 5% CO2, before removal of extracellular parasites by washing five times with serum-free RPMI. Infected THP-1 cells were then incubated for variable lengths of time, washing three times daily with serum-free RPMI, before washing with PBS and subjecting cells to methanol fixation and Giemsa staining. At least 100 macrophages were counted per sample, and a total of four independent biological replicates were used.

### Sample preparation for metabolomics

For both GC-MS and LC-MS metabolomic analyses the sample extraction was performed following the method described previously [56]. Briefly, 10^8^ cells were used for a final 200 μl sample. Cells were rapidly cooled in a dry ice/ethanol bath to 4°C, centrifuged, washed with 1 x PBS, and resuspended in extraction solvent (chloroform:methanol:water, 1:3:1 volume ratio). Following shaking for 1 h at 4°C, samples were centrifuged at 16,000g, 4°C for 10 min and the obtained supernatant was collected and stored under argon atmosphere at -80°C until the analysis. For medium analysis, 10 μl of medium was mixed with 190 μl extraction solvent. For the experiments involving labelling, cells were cultivated in Homem medium containing 50% of U-^13^C-glucose (Cambridge Isotope Laboratories, Inc.), or 100% 1,2-^13^C-glucose (Cambridge Isotope Laboratories, Inc.).

### LC-MS

The analyses were performed using separation on 150 x 4.6 mm ZIC-pHILIC (Merck) on UltiMate 3000 RSLC (Thermo Scientific) followed by mass detection on an Orbitrap Exactive mass spectrometer (Thermo Fisher) at Glasgow Polyomics. Analyses were performed in positive and negative polarity switching mode, using 10 μl injection volume and a flow rate of 300 μl/min. The samples were run alongside 249 authentic standards at 10 μM each. The data were processed and analyzed using mzMatch software [57], IDEOM [58], mzMatchISO [59], and MetaboAnalyst [60]. All the MS analyses were performed in 4 replicates, means of which are indicated. For the studies involving U-^13^C-glucose labelling, non-labelled samples were run in parallel and the natural labelling subtracted. Metabolites identified based on match with respective standards are indicated, the rest is predicted based on mass and retention time. Metabolomics data have been deposited to the EMBL-EBI MetaboLights database (10.1093/nar/gks1004. PubMed PMID: 23109552) with the identifier MTBLS491.

### Sample derivatisation for GC-MS

Sample derivatisation, analysis and data processing were performed at Glasgow Polyomics and reported previously [61]. A retention index mix was prepared from pure alkanes dissolved in hexane to final concentration of 6 mg/ml. Stock solutions at 1 mM were prepared for each from neat reference standard in water. A custom standard mixture of sugars, sugar phosphates, and pentose phosphate pathway intermediates were then prepared by mixing the stock solutions together and diluting with water until an acceptable peak was observed during GC-MS analysis.

30 μl of extracted sample, as well as each standard mix of sugars, sugar phosphates, and pentose phosphate pathway, were transferred into a 300 μl KIMSHIELD deactivated glass polyspring insert (National Scientific). Internal standards ^13^C_6_-Glucose (2 nmol), D_27_-Myristic Acid (2 nmol) and Scyllo-Inositol (1 nmol) were added to each sample. Samples were dried in a Savant SPD1010 SpeedVac concentrator (Thermo Scientific) for 90 min. Inserts were placed into a 9 mm screw cap amber borosilicate glass 1.5 ml vial (Thermo Scientific). 50 μl of 20 mg/ml (w/v) methoxyamine HCl in pyridine was added to each dried sample and sealed. The vial and insert were vortexed for 10 seconds and incubated at 60°C for 120 min. Following the methoximation step, 90 μl of N-Methyl-N-(trimethylsilyl) trifluoroacetamide + 1% trimethylchlorosilane was added, followed by a further 10 second of vortexing. Silylation was performed by incubation at 80°C for a further 120 min. Samples were cooled to room temperature. 10 μl of retention index alkane mixture was added to each sample. Samples were then ready for injection.

### GC-MS analysis

1μl of derivatized sample was injected into a Split/Splitless (SSL) injector at 280°C using a 1 in 50 split flow using a Trace 1310 gas chromatograph (Thermo Scientific). Helium carrier gas at a flow rate of 1.2 ml/min was used for separation on a TraceGOLD TG-5SILMS 30 m length × 0.25 mm inner diameter × 0.25 μm film thickness column (Thermo Scientific). The initial oven temperature was held at 70°C for 4 min, followed by an initial gradient of 20°C/min ramp rate. The final temperature was 320°C and held for 5 min. Eluting peaks were transferred through an auxiliary transfer temperature of 250°C into a GC-Q-Exactive mass spectrometer (Thermo Scientific). Electron impact (EI) ionisation at 70 eV energy, emission current of 50 μA with an ion source of 230°C. A filament delay of 5.3 min was used to prevent excess reagents from being ionised. High resolution EI fragment spectra were acquired using 60,000 resolution with a mass range of 50–650 *m/z*. The best internal lock mass from 207.0324, 281.0511 or 355.0699 *m/z* was used to maintain mass accuracy throughout the chromatogram.

### IC-MS/MS

WT and Δtkt mutants of *L*. *mexicana* were sampled by a fast filtration method as reported in Stoffel *et al*. [22]. Total sampling time was below 8 s. The extraction of intracellular metabolites was carried out by transferring the filters containing the pellets into 5 ml of boiling water for 30 s. 200 μl of a uniformly ^13^C-labeled *E*. *coli* cell extract was added as quantification internal standard [62], and the mixture was briefly vortexed (≈2 s). The extracts were immediately filtered (0.2 μm) and chilled with liquid nitrogen. After lyophilisation, the dried extracts were resuspended in 200 μl Milli-Q water prior to analysis. Three replicates were taken from each culture media, sampled and analysed separately. Metabolites involved in different parts of the metabolic network of *L*. *mexicana* cells were determined by liquid ion chromatography coupled to tandem mass spectrometry as described [22] with measured concentrations of metabolites expressed as a total cellular concentration assuming a volume of 10^8^ cells being equal to 5.8 μl.

### RNA sequencing

For RNA sequencing the RNA was isolated using RNeasy Mini Kit (Qiagen). The sequencing was performed at Glasgow Polyomics on the NextSeq500 platform, using library preparation by polyA selection, paired-end samples with 13 million reads per sample. The analysis was performed using the Galaxy interface [63,64], Bowtie2 software [65] and Cufflinks package [66].

### Enzyme activities

To measure enzyme activities on cell extract, 2 x 10^7^ cells were used for 100 μl of final extract. Cells were centrifuged, washed with 1 x PBS, and resuspended in TE lysis buffer (10 mM Tris-HCl pH 8.0, 1mM EDTA) with 0.15% Triton X-100 and Complete protease inhibitor cocktail (Roche). After 20 min incubation, the lysates were centrifuged (13,000 g, 16°C, 10 min) and supernatant collected. All the reactions were coupled to another enzyme, so the activity could be measured as production or consumption of NAD(P)H by UV-VIS Spectrophotometer (Shimadzu) at λ = 340 nm.

In order to measure hexokinase activity, the reaction mixture was prepared containing 300 mM Tris-HCl pH 7.5, 25 mM NaCl, 3 mM glucose, 2 mM ATP, 2 mM MgCl_2_, 1mM NADP, and 1 U G6PDH (all from Sigma-Aldrich) in 1ml total volume. The reaction mixture for glucose-6-phosphate isomerase contained 100 mM triethanolamine pH 7.5, 7mM MgCl_2_, 1.3 mM F6P, 0.4 mM NADP, and 1U G6PDH (all from Sigma-Aldrich) in 1 ml total volume. The reaction mixture for fructose-1,6-bisphosphate aldolase contained 100 mM triethanolamine pH 7.6, 2 μM EDTA, 10 μM fructose 1,6-bisphosphate, 1 U triosephosphate isomerase, 1 U glycerol-3-phospahte dehydrogenase and 100 μM NADH (all from Sigma-Aldrich) in 1 ml total volume. For 6PGDH measurement, the reaction contained 50 mM triethanolamine pH 7.5, 5mM MgCl_2_, 2 mM 6-phosphogluconate and 1 mM NADP (all from Sigma-Aldrich). When adding 1 mM glucose 6-phosphate into that mixture, the sum of activities of G6PDH and 6PGDH was measured and G6PDH activity was subsequently calculated from the difference.

### GFP-TKT constructs

The GFP-TKT construct was made using pNUS-GFPnH vector ([67] (http://www.ibgc.u-bordeaux2.fr/pNUS/greenvectors.html). The following primers were used: 5’- TAAGATCTATGGCCTCCATTGAGAAGGTGG—3’ and 5’- TACTCGAGTTACATCTTGCTGAATGAAGA– 3’, restriction sites for *Bgl*II and *Xho*I are underlined. For the TKT constructs with elongated and shortened C-termini, dsDNA constructs of the respective sequences were purchased from GenScript, USA. The DNA fragments were cloned into pNUS-GFPnH vector harboring TKT gene using internal restriction site for *FspA*I. For the construct omitting the PTS1, the reverse primer 5’—TACTCGAGGAATGAAGAGTTCTTGAGCGGCGCC– 3’ was used.

### Immunofluorescence microscopy

For preparation of samples for immunofluorescence microscopy, 200 μl of cell culture was used for each sample. Cells were washed in 1 x PBS two times, resuspended and incubated in 1% formaldehyde (methanol free, Thermo Fisher) for 30 min. Triton X-100 was added to final 0.1% concentration, after 10 min incubation glycine was added to 0.1 M final concentration and incubated for 10 min. Samples were centrifuged at 1,300 g, for 10 min, resuspended in 200 μl of PBS, and spread on microscope slides. Subsequently, slides were washed in PBS and blocked in TB solution (1x PBS with 0.1% Triton X-100, 0.1% BSA) for 1 h at room temperature. The primary α-TIM antibody was added in 1:100 dilution (kindly provided by Prof. Paul Michels, University of Edinburgh) in TB solution and incubated at 4°C overnight. Slides were washed three times in 1 x PBS, and incubated with secondary antibody (AlexaFluor 594 anti-rabbit, 1:1000, Molecular Probes) for 1 h at room temperature. Slides were washed three times in 1 x PBS, subsequently mounted with 2.5 μM DAPI, and covered with cover slides. Cells were visualised using Axioplan2 (Zeiss) and Volocity software.

### Glucose consumption

To estimate glucose consumption by *Leishmania* parasites, cultures were started at 10^7^ cells/ml density in fresh Homem medium containing only 1 mM glucose plus 10% FCS. At respective time points cell density was counted and a sample of spent media taken from each culture flask. Medium was kept at -20°C until the glucose concentration test was performed using GO Assay kit (Sigma-Aldrich) following the manufacturer’s instructions. Glucose consumption was calculated as reported previously [68].

### Analysis of cellular mannogen content by high performance thin layer chromatography (HPTLC)

Cellular mannogen content was visualised by HPTLC as previously described [69]. Briefly, 2.5 x 10^8^ mid-log phase cells were precipitated by centrifugation, washed once with PBS and then extracted for two hours in 1:2:0.8 chloroform:methanol:water at room temperature, agitating continuously at 1,500 rpm. Metabolite extracts were cleared by centrifugation at 17,000 g for 10 min and precipitated pellets were retained for protein content determination. Supernatants were dried in a Savant DNA 120 SpeedVac concentrator (Thermo) and resuspended in 1:1 1-butanol:water. After partitioning the aqueous layer was retained and further washed twice with an equal volume of water-saturated 1-butanol, then desalted using AG501-X8 resin (Bio-Rad). The aqueous phase was then dried and resuspended in 1:2:0.8 chloroform:methanol:water. HPTLC analysis was performed on aluminium-backed silica 60 plates (Merck), developing twice in 4:3:3 1-butanol:methanol:water, then visualising by spraying with orcinol/H_2_SO_4_ and heating at 100°C for 10 min. Protein content was determined by extracting precipitated pellets in 0.1 M NaOH at 4°C for 30 min with continuous shaking at 1,500 rpm, centrifugation at maximum speed for 10 min then measuring protein concentration using the Bio-Rad protein assay dye reagent (Bio-Rad) in comparison to a bovine serum albumin standard curve. Quantification of mannogen was performed using ImageJ, based on three biological replicates and normalised per protein content.

## Supporting information

S1 Fig Schematic representation of the TKT locus replacement. (PDF)

S2 Fig Labelling pattern for octulose 8-phosphate. (PDF)

S3 Fig Amino acids detected in spent medium metabolomics analysis. (PDF)

S4 Fig Phenotypic characterisation of cells expressing solely cytosolic or solely glycosomal TKT. (PDF)

S5 Fig LC-MS metabolomics comparison between Δtkt + cyto GFP-TKT and Δtkt + glyco GFP-TKT. (PDF)

S6 Fig Alignment of fructose-1,6-bisphosphate aldolase protein sequences. (PDF)

S1 Table IDEOM file of LC-MS analysis of WT, Δtkt, and Δtkt + TKT cells. (XLSB)

S2 Table The complete dataset of GC-MS analysis of WT and Δtkt cells. (XLSX)

S3 Table The complete dataset of RNAseq analysis of WT, Δtkt, and Δtkt + TKT cells. (XLSX)
