# Supplementary material for: Deletion of transketolase triggers a stringent metabolic response in promastigotes and loss of virulence in amastigotes of Leishmania mexicana
Source: PLoS Pathog. 2018 Mar 19;14(3):e1006953. doi: 10.1371/journal.ppat.1006953 (PMC5882173; doi:10.1371/journal.ppat.1006953)
Supplement: S1 Fig — (PDF) [file ppat.1006953.s001.pdf]

S1 Fig. Schematic representation of the TKT locus replacement. The ORFs are indicated by a solid black arrow, the TKT flanking regions are indicated by striped boxes, and the restriction sites are indicated by vertical lines. (A) Endogenous TKT locus. (B) TKT locus upon successful integration of the hygromycin (HYG) knock-out cassette. (C) TKT locus upon successful integration of the nourseothricin (SAT) knock-out cassette. (D) Screen for internal transcribed spacer 1 region to generate an expected product of 350 bp to confirm presence of *Leishmania* genomic DNA. (E) Screen for TKT to generate an expected product of 2100 bp. The clone in lane 2 was selected for further experiments. (F) Growth of WT, Δtkt, and Δtkt + GFP-TKT axenic amastigote cells.

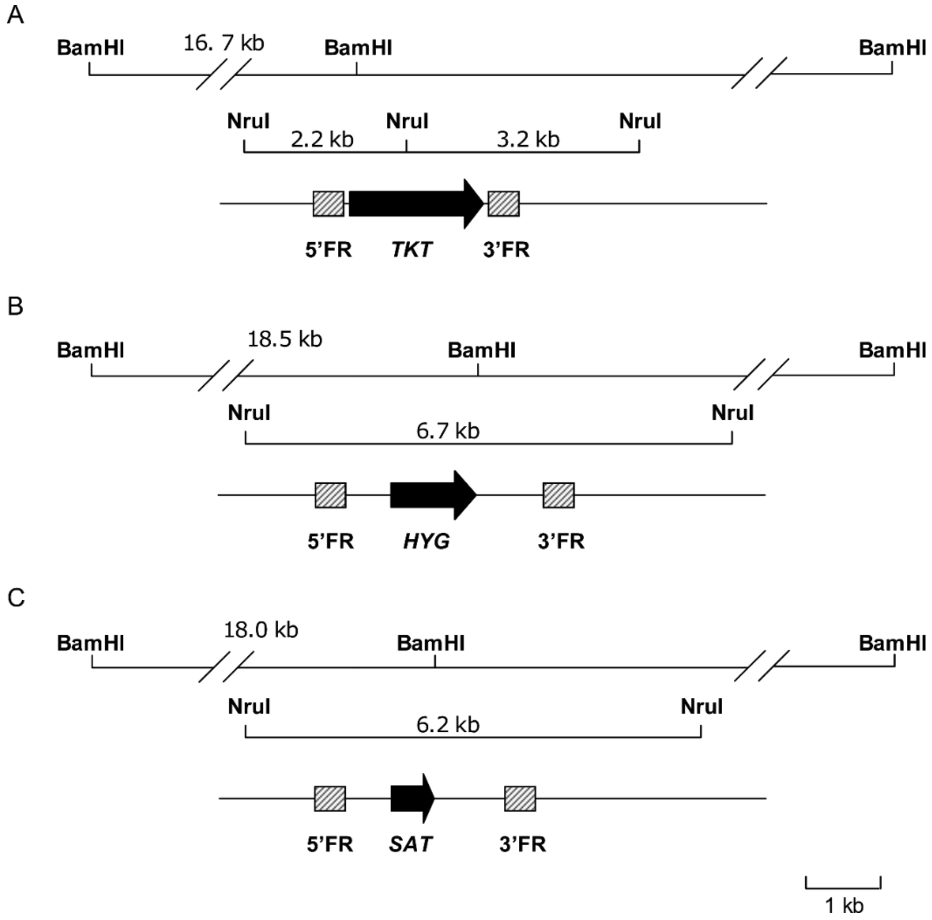

D

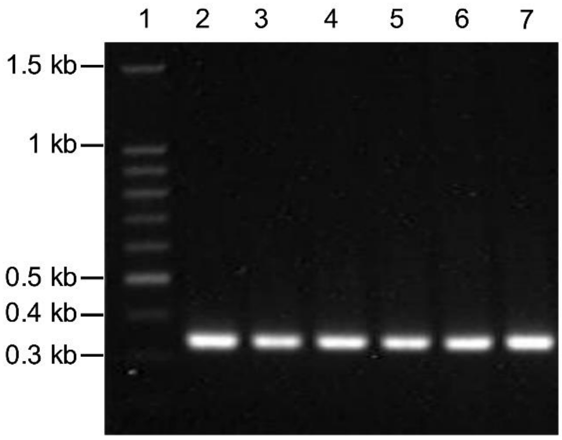
1 2 3 4 5 6 7
1.5 kb
1 kb
0.5 kb
0.4 kb
0.3 kb

E

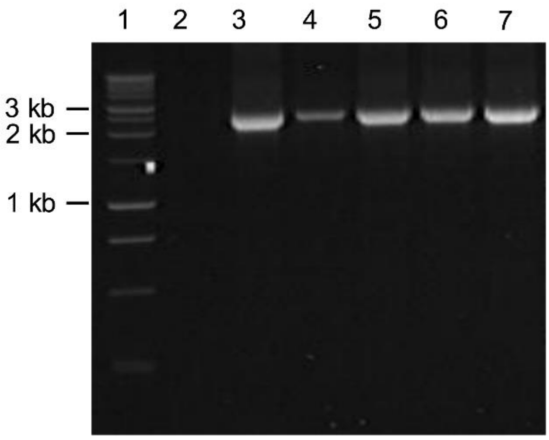
1 2 3 4 5 6 7
3 kb
2 kb
1 kb

F

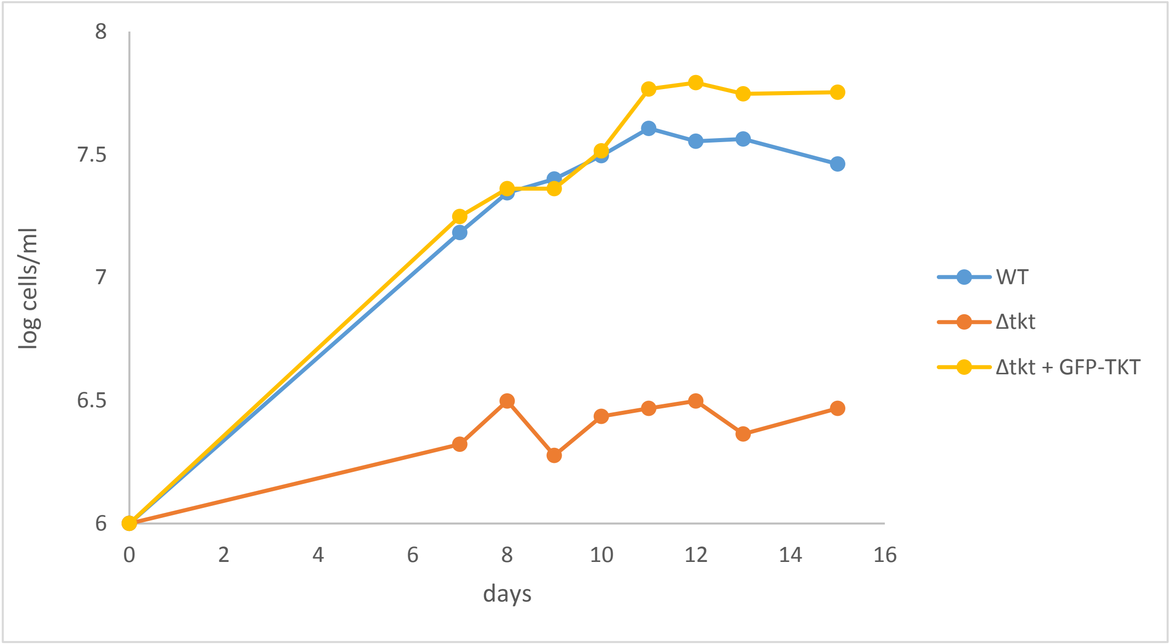
log cells/ml
8
7.5
7
6.5
6
0 2 4 6 8 10 12 14 16
days
WT
Δtkt
Δtkt + GFP-TKT
