# Supplementary material for: Deletion of transketolase triggers a stringent metabolic response in promastigotes and loss of virulence in amastigotes of Leishmania mexicana
Source: PLoS Pathog. 2018 Mar 19;14(3):e1006953. doi: 10.1371/journal.ppat.1006953 (PMC5882173; doi:10.1371/journal.ppat.1006953)

S3 Fig. Amino acids detected in spent medium metabolomics analysis. White – fresh medium, black – WT, grey – Δtkt.

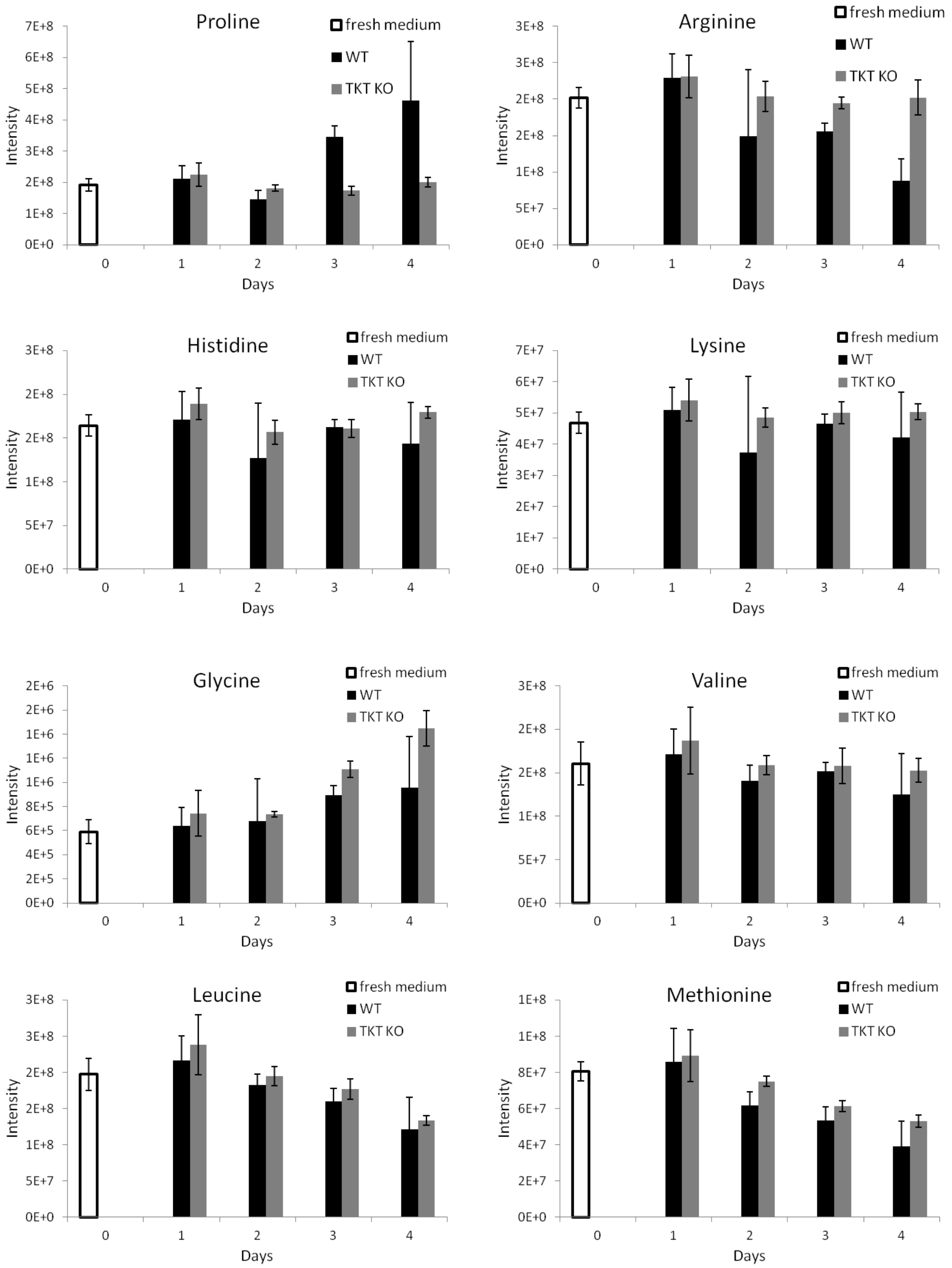

Supplement: S3 Fig — (PDF) [file ppat.1006953.s003.pdf]
