# Supplementary material for: Deletion of transketolase triggers a stringent metabolic response in promastigotes and loss of virulence in amastigotes of Leishmania mexicana
Source: PLoS Pathog. 2018 Mar 19;14(3):e1006953. doi: 10.1371/journal.ppat.1006953 (PMC5882173; doi:10.1371/journal.ppat.1006953)
Supplement: S4 Fig — (PDF) [file ppat.1006953.s004.pdf]

S4 Fig. Phenotypic characterisation of cells expressing solely cytosolic or solely glycosomal TKT. (A) Growth curves. (B) Alamar Blue Assay with glucose oxidase (GOX), fluorescence detected corresponds with viability of cells. Error bars indicate standard deviation, n = 3. (C) Western blot analysis of cells expressing GFP-TKT variants probed with α-GFP antibody.

A

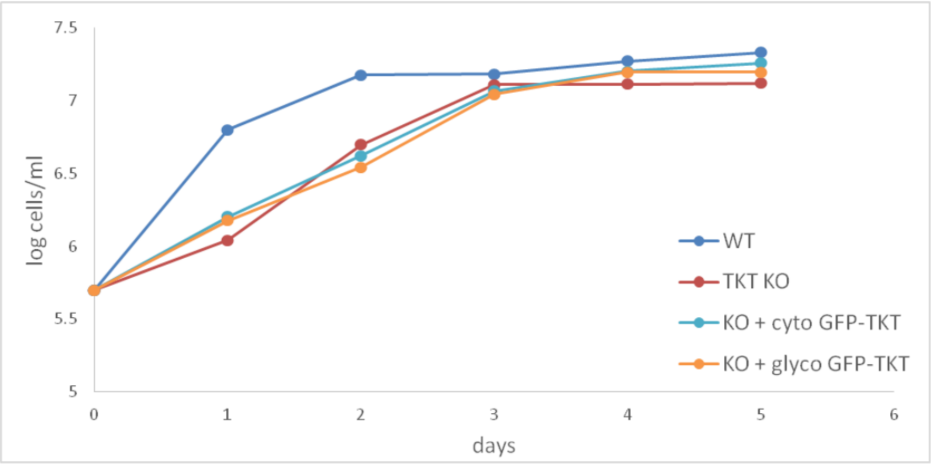

B

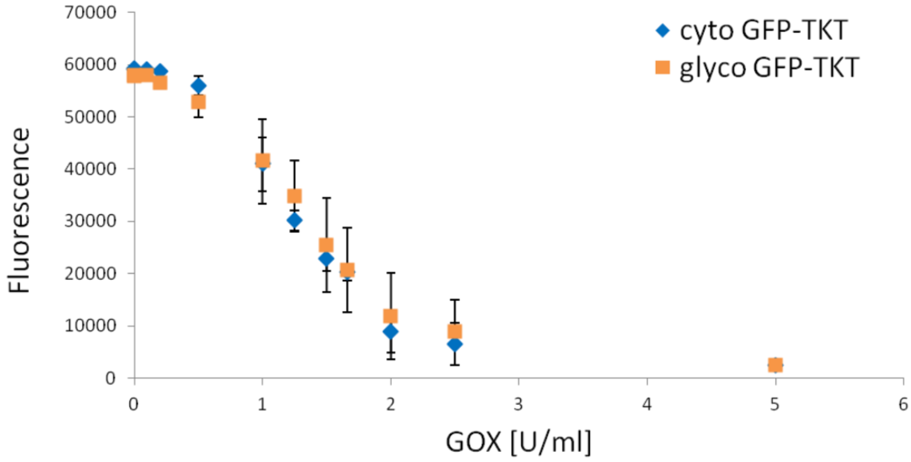

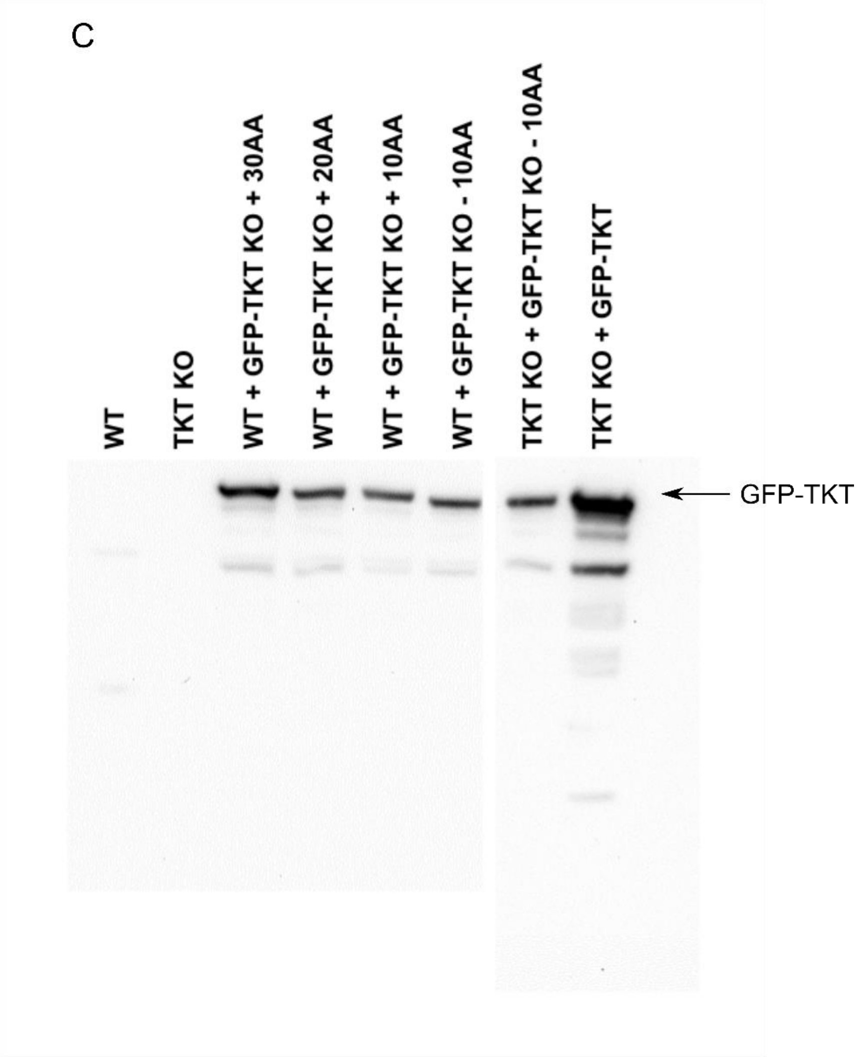
C
WT
TKT KO
WT + GFP-TKT KO + 30AA
WT + GFP-TKT KO + 20AA
WT + GFP-TKT KO + 10AA
WT + GFP-TKT KO - 10AA
TKT KO + GFP-TKT KO - 10AA
TKT KO + GFP-TKT
GFP-TKT
