# Supplementary material for: Deletion of transketolase triggers a stringent metabolic response in promastigotes and loss of virulence in amastigotes of Leishmania mexicana
Source: PLoS Pathog. 2018 Mar 19;14(3):e1006953. doi: 10.1371/journal.ppat.1006953 (PMC5882173; doi:10.1371/journal.ppat.1006953)

S5 Fig. LC-MS metabolomics comparison between Δtkt + cyto GFP-TKT and Δtkt + glyco GFP-TKT. (A) Selected metabolites of glycolysis and the PPP, the numbers represent relative abundance compared to WT. (B) Heat map of all the metabolites detected, yellow indicates no change, red increase and green decrease. The complete data are included in Tab S1.

A

	WT	TKT KO	KO + endog TKT	KO + GFP-TKT	KO + cyto GFP-TKT	KO + glyco GFP-TKT
Glucose	1.00	4.79	1.13	2.08	1.80	1.96
Glucose 6P/ Fructose 6P	1.00	0.75	0.77	**0.56**	0.62	**0.54**
6-Phosphogluconate	1.00	**5.23**	0.58	0.44	0.47	0.45
Pentose P	1.00	**212.06**	0.81	0.72	0.98	0.85
Sedoheptulose 7P	1.00	**0.31**	0.77	0.78	0.72	0.73
Octulose 8P	1.00	**230.02**	0.77	**0.62**	1.01	0.64
PEP	1.00	**0.14**	**0.67**	**0.59**	**0.63**	0.72
Mannogen	1.00	**0.10**	**0.18**	**0.13**	**0.14**	**0.17**
Fructose 1,6-bisP	1.00	**0.42**	**0.53**	**0.30**	**0.43**	**0.32**
Ribose 1,5-bisP	1.00	**2.58**	**1.65**	0.60	0.96	**0.44**

B

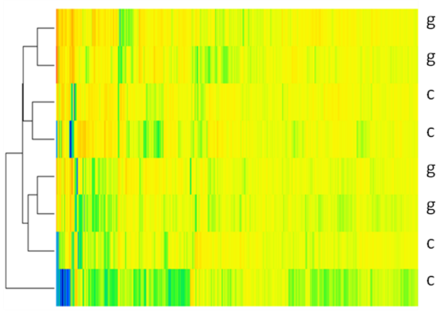

Supplement: S5 Fig — (PDF) [file ppat.1006953.s005.pdf]
