# Supplementary material for: Deletion of transketolase triggers a stringent metabolic response in promastigotes and loss of virulence in amastigotes of Leishmania mexicana
Source: PLoS Pathog. 2018 Mar 19;14(3):e1006953. doi: 10.1371/journal.ppat.1006953 (PMC5882173; doi:10.1371/journal.ppat.1006953)
Supplement: S6 Fig — (PDF) [file ppat.1006953.s006.pdf]

S6 Fig. Alignment of fructose-1,6-bisphosphate aldolase protein sequences from *Arabidopsis thaliana* (Q944G9), human (PO4075), and *L. mexicana* (LmxM.36.1260). Representatives of the enzymes reported to be inhibited by PPP intermediates and *L. mexicana* sequences are shown. Arrows indicate the conserved amino acid residues crucial for substrate binding and activity.

Unconserved 0 1 2 3 4 5 6 7 8 9 10 **Conserved**

```
             ..........10 ..........20 ..........30 ..........40 ..........50
bean Vigna   ---------- ---------- ---------- ---------- ----------
Arabidopsis  MASTSLLKAS PVLDKSEWVK GQSVLFRQPS SASVVLRNRA TSLTVR---A
human        ---------- ---------- ---------- ---------M PYQYPA---L
L. mexicana  ---------- ---------- ---------M SRVTV----F QSQLPAHNRL
Consistency  0000000000 0000000000 0000000000 1000100001 1221220002

             ..........60 ..........70 ..........80 ..........90 .........100
bean Vigna   -----DELIA NAAYIGTPGK GILAADESTG TIGKRLASIS VENVETNRRA
Arabidopsis  ASSYADELVK TAKTIASPGR GILAMDESNA TCGKRLDSIG LENTEANRQA
human        TPEQKKELSD IAHRIVAPGK GILAADESTG SIAKRLQSIG TENTEENRRF
L. mexicana  KTPYESELVA TVKKLTTPGK GLLAADESIG SCMKRFEPIG LSNTEEHRRQ
Consistency  121224**54 4743836**8 *8**6***47 754**746*7 57*7*47*73

             .........110.........120.........130.........140.........150
bean Vigna   LRELLFTTPG AFH-CLSGVI LFEETLYQNT ASGKPFVELL KEGGVLPGIK
Arabidopsis  FRTLLVSAPG -LGQYVSGAI LFEETLYQST TEGKKMVDVL VEQNIVPGIK
human        YRQLLLTADD RVNPCIGGVI LFHETLYQKA DDGRPFPQVI KSKGGVVGIK
L. mexicana  YRALMLEAEG -LEQYISGVI LHDETVGQKA ANGQTFPEYL TTRGVVPGIK
Consistency  5*4*855746 0532487*7* *65**85*56 45*6474658 454648 6***

             .........160.........170.........180.........190.........200
bean Vigna   VDKGTVEL-A GTNGETTTQG LDGLGQRCQK YYEAGARFAK WRAVLKIGPN
Arabidopsis  VDKGLVPL-V GSNNESWCQG LDGLSSRTAA YYQQGARFAK WRTVVSI-PN
human        VDKGVVPL-A GTNGETTTQG LDGLSERCAQ YKKDGADFAK WRCVLKIGEH
L. mexicana  TDMGLCPLLE GAEGEQMTEG LDGYAKRASA YYKKGCRFCK WRNVYKIQNG
Consistency  7*6*566*04 *676*5368* ***655*454 *663*66*6* **3*57*244

             .........210.........220.........230.........240.........250
bean Vigna   EPSELAIHEN AYGLARYAAI CQENGLVPIV EPEILVDGRH DINKCAAVTE
Arabidopsis  GPSALAVKEA AWGLARYAAI SQDSGLVPIV EPEILLDGEH DIDRTYDVAE
human        TPSALAIMEN ANVLARYASI CQQNGIVPIV EPEILPDGDH DLKRCQYVTE
L. mexicana  TVSEAAVRFN AETLARYAIL SQMSGLVPIV EPEVMIDGKH DIDTCQRVSE
Consistency  36*56*9356 *23*****58 5*47*8**** ***984**4* *855642*6*

             .........260.........270.........280.........290.........300
bean Vigna   RVLAACYKAL NDHHVLLEGT LLKPNMVTPG SESP-KVTPE VIAQYTVAAL
Arabidopsis  KVWAEVFFYL AQNNVMFEGI LLKPSMVTPG AESKDRATPE QVAAYTLKLL
human        KVLAAVYKAL SDHHIYLEGT LLKPNMVTPG HACTQKFSHE EIAMATVTAL
L. mexicana  HVWREVVAAL QRHGVIWEGC LLKPNMVVPG AESGKTATPE QVAHYTVMTL
Consistency  5*465653 6* 4474944**4 ****7**7** 466225475* 59*36*835*

             .........310.........320.........330.........340.........350
bean Vigna   QRTVPAAVPA IVFLSGGQSE EEATLNLNAM NKLQGKKPWS LSFSFGRALQ
Arabidopsis  RNRVPPAVPG IMFLSGGQSE VEATLNLNAM NQAP--NPWH VSFSYARALQ
human        RRTVPPAVTG ITFLSGGQSE EEASINLNAI NKCPLLKPWA LTFSYGRALQ
L. mexicana  ARTMPAMLPG VMFLSGGLSE VQASEYLNAI NKSPLPRPYF LSFSYARALQ
Consistency  5767*56867 95*****6** 48*745***7 *736115*73 87**86****

             .........360.........370.........380.........390.........400
bean Vigna   QSTLKAWGGK DENIKKAQDA FIARCKANSQ ATLATYKGDA TLSEGASESL
Arabidopsis  NTCLKTWGGR PENVNAAQTT LLARAKANSL AQLGKYTGEG E-SEEAKEGM
human        ASALKAWGGK KENLKAAQEE YVKRALANSL ACQGKYTPSG QAGAAASESL
L. mexicana  SSALKAWGGK ESGVPAGRRA FLHRARMNSL AQLGKYKRAD --DDASTSSL
Consistency  475**7***8 3777467734 574*656**6 *3676*5344 2055485778

             .......
bean Vigna   HVKNYKY
Arabidopsis  FVKGYTY
human        FVSNHAY
L. mexicana  YVKGNTY
Consistency  5*7644*
```
